# Tools for Single-Cell Kinetic Analysis of Virus-Host Interactions

**DOI:** 10.1371/journal.pone.0145081

**Published:** 2016-01-11

**Authors:** Jay W. Warrick, Andrea Timm, Adam Swick, John Yin

**Affiliations:** Systems Biology Theme, Wisconsin Institute for Discovery, Department of Chemical and Biological Engineering, University of Wisconsin-Madison, Madison, WI, United States of America; International Centre for Genetic Engineering and Biotechnology, ITALY

## Abstract

Measures of cellular gene expression or behavior, when performed on individual cells, inevitably reveal a diversity of behaviors and outcomes that can correlate with normal or diseased states. For virus infections, the potential diversity of outcomes are pushed to an extreme, where measures of infection reflect features of the specific infecting virus particle, the individual host cell, as well as interactions between viral and cellular components. Single-cell measures, while revealing, still often rely on specialized fluid handling capabilities, employ end-point measures, and remain labor-intensive to perform. To address these limitations, we consider a new microwell-based device that uses simple pipette-based fluid handling to isolate individual cells. Our design allows different experimental conditions to be implemented in a single device, permitting easier and more standardized protocols. Further, we utilize a recently reported dual-color fluorescent reporter system that provides dynamic readouts of viral and cellular gene expression during single-cell infections by vesicular stomatitis virus. In addition, we develop and show how free, open-source software can enable streamlined data management and batch image analysis. Here we validate the integration of the device and software using the reporter system to demonstrate unique single-cell dynamic measures of cellular responses to viral infection.

## Introduction

Phenotypic cellular heterogeneity arises due to myriad intrinsic and extrinsic factors and represents a topic of growing importance in biology. Intrinsic factors represent genetic or epigenetic alterations, while extrinsic factors include neighboring cells, the extracellular matrix, or the organism physiology. Cell heterogeneity impacts disease, including the development of cancer and drug resistance [1, 2] as well as normal biology, including activation of primary and secondary immune responses [3–5] and of developmental processes [6, 7]. Furthermore, heterogeneity exists even under tightly controlled and homogeneous conditions such as the culture of a clonogenic cell-line in a standard culture flask [8, 9]. Single-cell quantification of such heterogeneity (cytometry) represents a unique opportunity to detect and discover naturally arising correlations among cellular characteristics, yielding new insights that would be more challenging or impossible to gain using population-average measures [10]. Complicating this opportunity, however, is the dynamic nature of of cellular behaviors. While overall distributions of cell phenotypes in a population might appear relatively constant, the characteristics of individuals are constantly in flux [11].

Dynamic cytometry (DC), or the ability to measure the time-dependent behavior of individual cells within a heterogeneous population, can help address this challenge. Fundamentally, DC enables insight into areas of biology where heterogeneity and dynamics are important or where rare events are hidden by population averages. For this reason, dynamic cytometry is particularly well-suited for the study of virus-host interactions, where infection and signaling can involve stochastic events and variable dynamics [3, 12–19].

**Fig 1:**
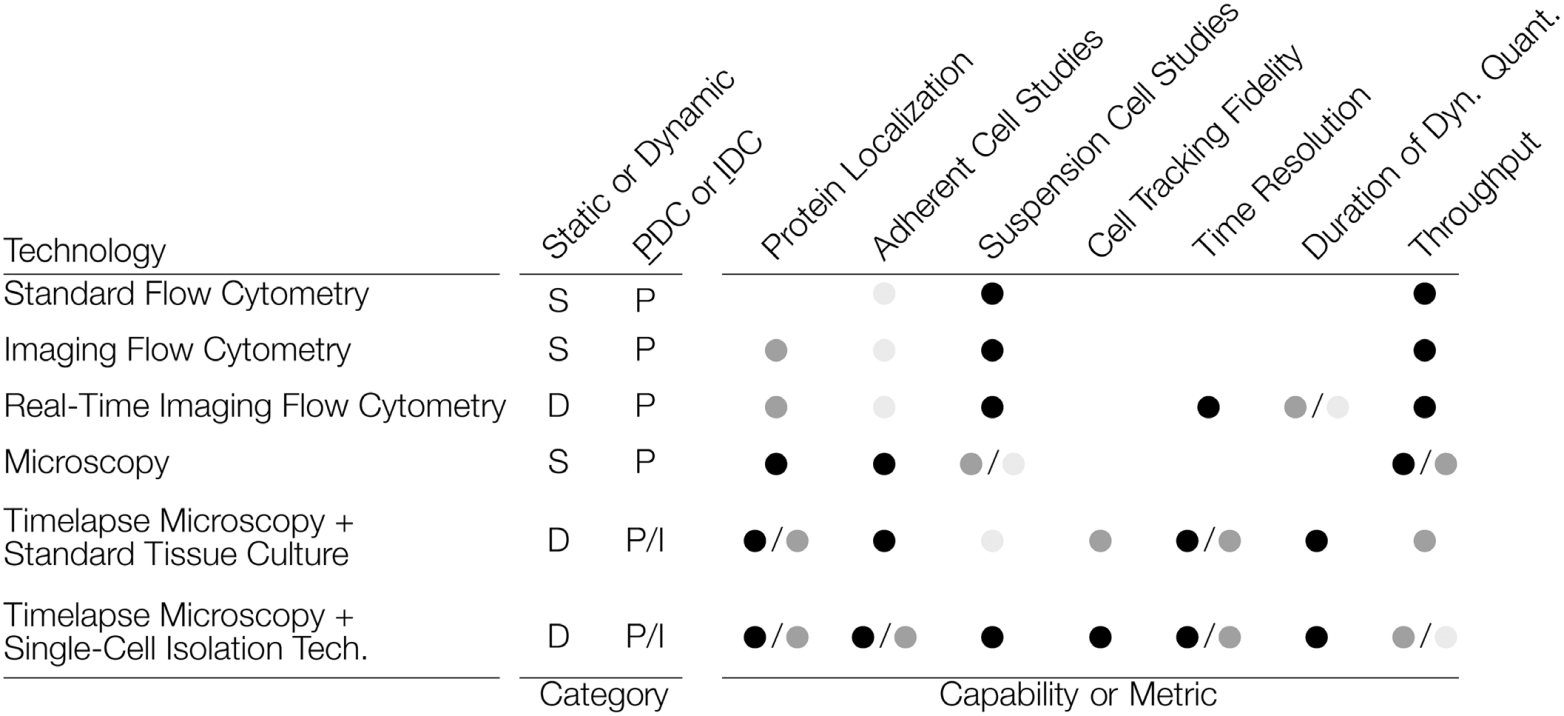
Comparison of general approaches that enable fluorescence-based cytometry. The categories and capabilities of each are roughly assessed. Capability Key: ● = High, ● = Medium, ● = Low, (blank) = not significant.

Viewed broadly, DC methods can be described as those that quantify population distributions over time (population dynamic cytometry, PDC), and those that track or follow individual cells over time (individual dynamic cytometry, IDC). Fig 1 compares common methods for static, dynamic, population, and individual cytometry methods. Although PDC approaches can enable new insights into cellular dynamics, there remain many fundamental questions to be answered that require IDC. For example, relatively little is known about how population distributions are formed and maintained by the constantly changing individual cells that make up those distributions. Likewise, IDC enables one to link the kinetics of highly heterogeneous and stochastic events within individual cells, such as virus-host interactions during an infection, which could provide significant new insights. Such applications are enabled by the ability to identify and follow individual cells through time.

There exist multiple approaches to enabling IDC such as cellular bar-coding, image-based cell tracking analysis, and single-cell isolation [20–27]. Although no single approach is ideal for all applications, cell confinement strategies can offer an attractive balance of advantages and limitations. Cellular bar-coding methodologies have tremendous potential to impact a variety of fields including *in vivo* work; however, in the context of high-throughput IDC, it is difficult to implement a cellular bar-coding scheme that provides a sufficient number of labels, does not impact the biology, and is simple to implement compared to alternative identification methods. Likewise, image analysis software for tracking and quantifying cells (*e.g.*, via image segmentation) can be a powerful and cost-effective technique for IDC but encounters limits when performing extended single-cell kinetics studies (*i.e.*, studies that generate a contiguous time-series of data for each cell over many timepoints). In such cases, tracking must be able to identify cells and/or their boundaries with ∼ 100% accuracy from timepoint-to-timepoint to provide contiguous information for kinetics analysis. For example, even with 99% tracking/identification accuracy between each timepoint, after 60 timepoints only 55% of the time-series collected will be complete and accurate for the full time-course of the experiment (0.99^59^, which assumes independence that does not hold in practice, yet illustrates the general problem that arises as the number of timepoints increases). Given that cells can move, change shape, divide, lyse, and may appear to overlap; robust determination of cell identity among adjacent or overlapping cells is often not feasible via software or by the human eye. These challenges and considerations are quantified and described in more detail in previous work by others.[28–30]

In contrast, cell isolation or confinement strategies (*e.g.*, microfluidic entrapment, microwell arrays, droplet-based microfluidics, digital microfluidics, and adhesion patterning) offer the ability to encode the identity of a cell via its location, which makes tracking and quantification of cells in images unambiguous and simple. Furthermore, this can be done for roughly hundreds of thousands of cells per device or ‘chip’. However, some drawbacks are that cells cannot migrate, spatially organize or interact, as they might *in vivo* or in un-constrained culture. Likewise, experimental complexity is generally increased to different degrees depending on the method used to isolate or confine the cells. Additionally, IDC often produces extremely large datasets and associated challenges of data management/organization, batch processing, and data analysis that should not be ignored.

In principle, IDC can enable significant opportunity to extract insight from measures of cell heterogeneity. However, use of IDC approaches remains limited. To expand its use, IDC will need to simplify and integrate: 1) dynamic readouts of cellular processes, 2) robust methods for following cells over extended times, and 3) software for streamlined data management and analysis. Furthermore, IDC has specific potential to impact the study of virus-host interactions where variability, stochasticity, and dynamics are pushed to an extreme. Here we describe and validate a microscopy-based approach to IDC that integrates a recently developed dual-color virus-host system for simultaneously studying virus progression and host response [31]; a new microwell-array (MA) embodiment for single-cell isolation, perturbation, and imaging; and new open-source software tools for more streamlined experimentation, analysis, and data management. The intent of this work is to validate the integrated approach for follow-on studies of the virus-host system, illustrate the potential for new insights into the dynamics of virus-host interactions, and establish advancements that simplify and streamline IDC, working towards methods that can be used by a broader research community.

## Methods

The integrated approach consists of three primary components: an engineered dual-color virus-host system, MAs for cell confinement, and open-source analysis and data management software.

### Dual-color virus-host system

The developed approach is demonstrated and validated using engineered strains of vesicular stomatitis virus (VSV) that express a red fluorescent protein (RFP), DsRed-Express DR, and a reporter cell line engineered to express a green fluorescent protein (GFP), Zs-Green, upon activation of cellular innate immunity. The reporter cells and fluorescent virus strains were created and characterized as described previously [31], and briefly summarized here.

**Fig 2:**
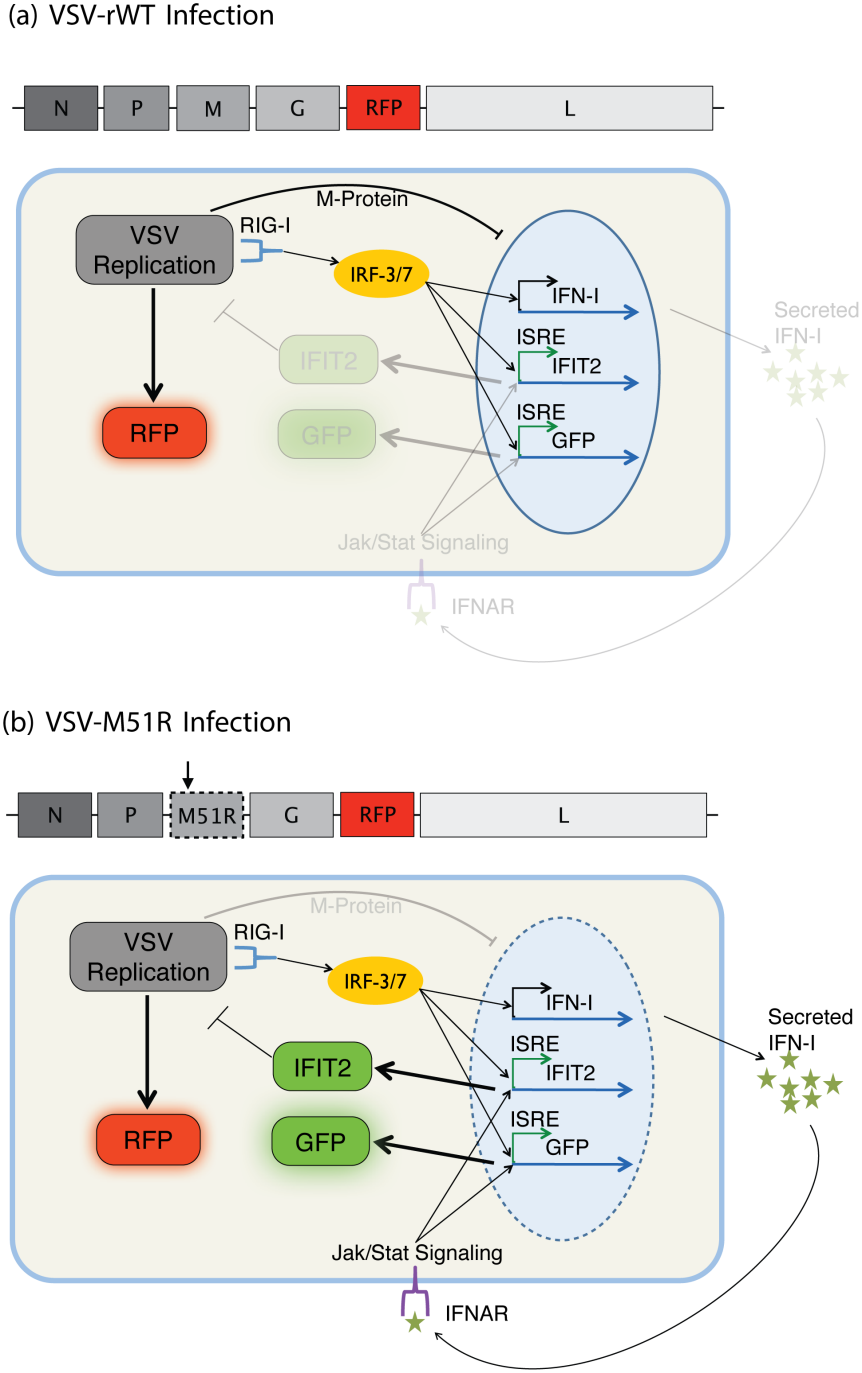
Infection with the recombinant VSV strains leads to the production of RFP as a reporter of viral protein production. (a) Although VSV-rWT does stimulate the early stages of immune recognition, its matrix (M) protein suppresses export of host transcripts, thereby suppressing the subsequent innate immune response and resulting in a GFP^−^RFP^+^cell. (b) The well characterized mutant of M protein, M51R [34], abolishes this function and readily stimulates the innate immune response [38]. Thus, an infection with VSV-M51R allows production of type-I interferons and activation of interferon stimulated genes, including the IFIT2 reporter, resulting in a GFP^+^RFP^+^cell.

VSV is a negative-sense, single-stranded, non-segmented RNA virus that is known for its broad cellular tropism, exceptionally fast replication kinetics in permissive cell types, sensitivity to interferon stimulated genes (ISGs), and frequent use in studies of innate immunity [32, 33]. Upon infection, VSV broadly prevents host-transcripts from being exported out of the nucleus via the viral M-protein, effectively disabling the innate immune response of the cell. However, a single point mutation in the M-protein (M51R) can disrupt this function, allowing transcript export and robust activation of innate immunity [34]. Engineered versions of the wild-type strain and M51R strain that contain the DsRed-Express DR gene in the 5^th^ gene position (VSV-rWT and VSV-M51R, respectively) were created to enable observation and comparison of viral protein production (Fig 2). The genes of VSV are transcribed in sequence off a single promoter with attenuation at each intergenic region. This results in defined ratios of gene transcripts that are determined by location along the viral genome [35, 36]. This characteristic enables us to use viral fluorescent protein production as a reporter of viral protein production. Placement of the fluorescent protein in the 5th gene position minimizes potential impact of the reporter expression on virus production while producing a robust fluorescent signal [31, 37]. RFP production is also correlated with the number of VSV plaque forming units (PFU), thus RFP is also an indicator of infectious virus particle production (see Fig A in S1 Text).

The reporter cell-line was developed using the PC3 human prostate cancer cell line which is known to have a robust innate immune response to infection by VSV due to an intact interferon pathway [39]. The reporter cell line was created via lentiviral insertion of an engineered gene followed by expansion from a single cell. The engineered gene encodes for the Zs-Green fluorescent protein and shares the promoter sequence of the human IFIT2 (interferon-induced protein with tetratricopeptide repeats 2) gene, a downstream reporter of interferon stimulation. Thus, upon promotion of the IFIT2 gene, the PC3 reporter cell line (PC3-IFIT2) produces GFP in addition to its native gene. Upon infection with the wild-type virus, VSV-rWT, cells robustly produce red fluorescence but little or no green fluorescence (i.e., the virus blocks the host response and GFP production). In contrast, infection with the mutant virus, VSV-M51R, produces robust red and green fluorescent protein (i.e., the virus is unable to block the host response or GFP production). IFIT2 is one of many interferon stimulated genes (ISGs). Thus, activation of IFIT2 indicates activation of a variety of ISGs with conserved promoter sequence motifs and is useful for indicating general activation of the host innate immune response. Experiments were performed with cells below passage 10 from stocking. Details of cell culture for these studies are contained in the S1 Text.

The virus and host were designed for each other, such that the protein maturation and degradation kinetics are rapid, well-matched, and have minimal impact on the salient features of the biological system [31] providing a pre-validated system for demonstrating our approach to IDC.

### Microwell arrays

**Fig 3:**
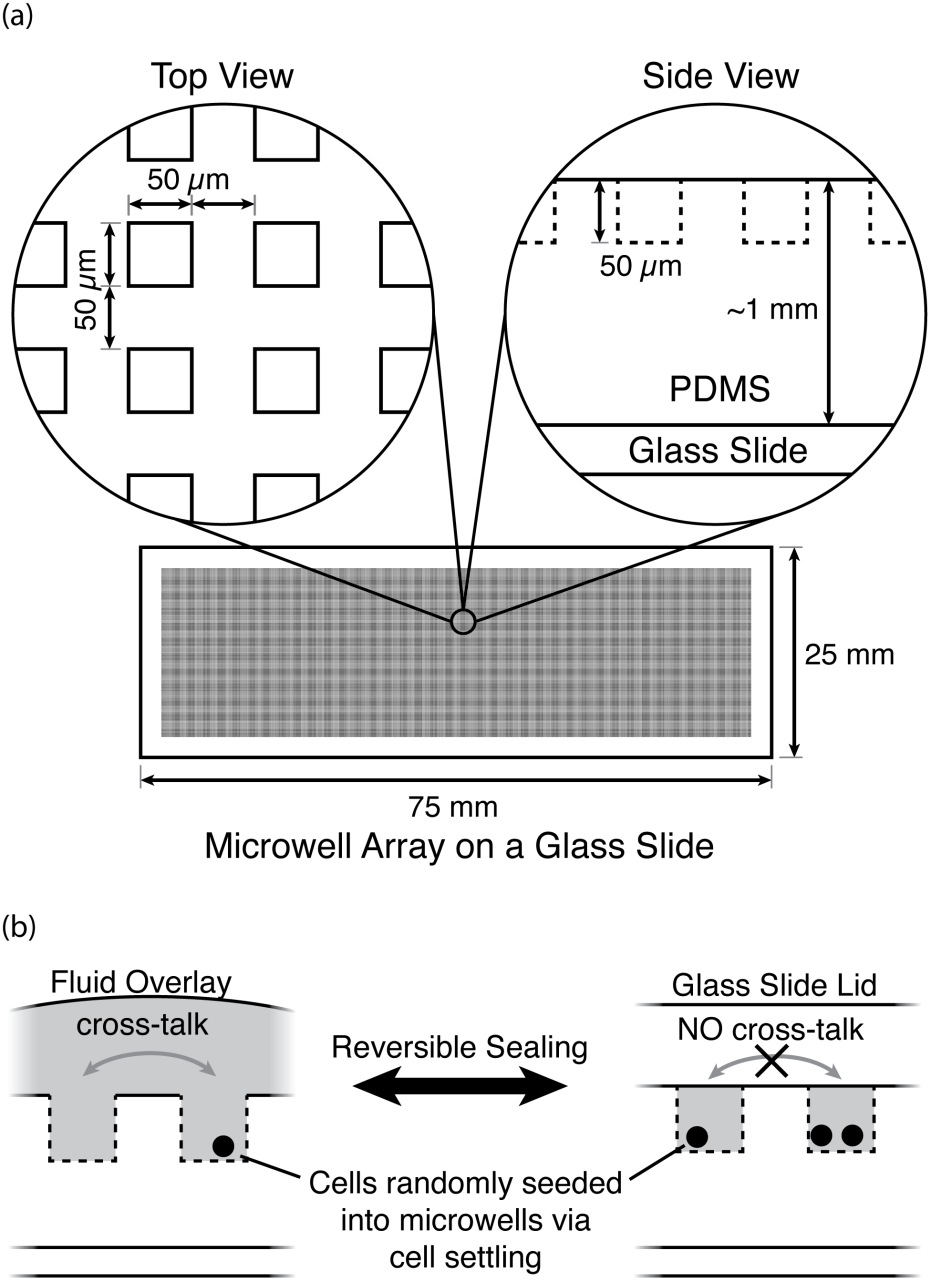
Traditional microwell array (MA) design. (a) Typical device dimensions. (b) A lid can be secured to the top of the PDMS MA to either allow or block cross-talk or communication between the microwells [46].

**Fig 4:**
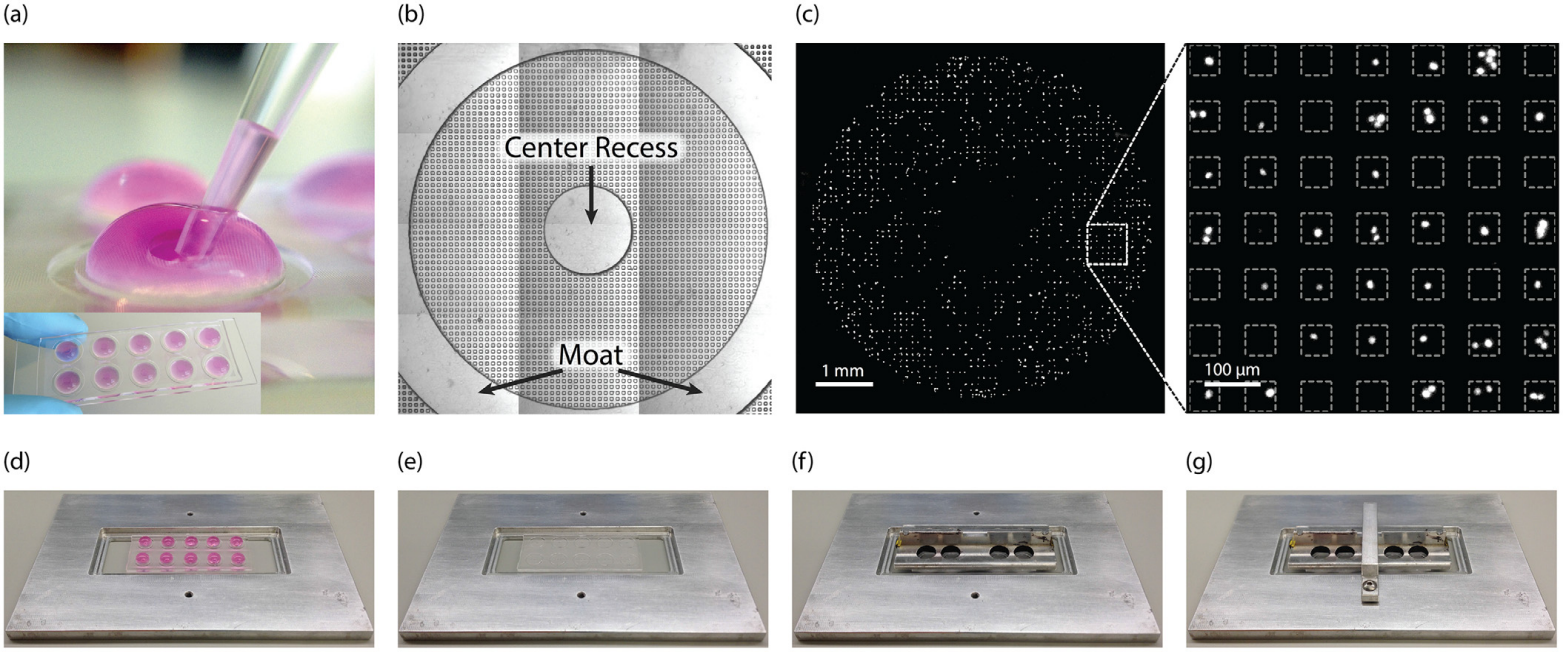
New microwell array (MA) device design and assembly for single-cell studies. (a) Regions of microwells are isolated from one another using grooves or ‘moats’ allowing multiple conditions to be tested on a single device using surface tension to maintain the droplets. Each isolated region is referred to as a bull’s-eye given its appearance. (inset, d, f) Twelve bull’s-eyes in a 2×6 array fit on a standard glass slide. The device is trimmed to include 2×5 and only the outer 8 can be imaged through the clamp device. (b-c) The array of bull’s-eyes containing ∼ 2500 microwells each are placed on a microscope slide. Cells in suspension within each droplet settle into microwells randomly for easy loading and are visualized using nuclear staining. Cells are identified and counted using image analysis. Only wells containing a single cell are considered. The image in (b) is a stitched phase contrast image, (c) is the same image with fluorescent nuclei visible, and the magnified image indicates the size and shape of individual microwells. (d-g) Device assembly. (d) The slide of bull’s-eyes is placed in the base of the microscope insert. (e) The droplets are removed and a glass slide is placed over the bull’s-eyes. (f) The pressure distributor of the microscope stage insert is placed over the glass slide lid. (g) The top plate is fastened with a bracket using two hex screws. The device is sealed and ready for time-lapse imaging.

The IDC method developed here uses single-cell isolation or entrapment to enable completely unambiguous long-term tracking of individual cells. Of the many potential strategies for isolating single cells (*e.g.*, limiting dilution, microfluidic flow-based cell entrapment, valve-based microfluidics, droplet-based microfluidics, single-cell aspiration), we chose to use microwell arrays (MAs, Figs 3 & 4) given their attractive balance of simplicity, throughput, and flexibility [40–45], characteristics which help facilitate statistically relevant datasets and adoption by others.

In established MA-based devices a cell suspension is typically overlaid on the MA, whereupon cells settle into individual microwells, thereby randomly isolating or entrapping single-cells. Similar to the process of cell seeding, treatments are performed using complete fluid overlays or by submerging the device, limiting the device to a single experimental condition [47] and requiring significant finesse [46] for repeatability and avoidance of spills, drips, and contamination. A common feature of these devices is that, at any point in time, a lid can be added or removed to control cellular cross-talk between microwells (Fig 3).

Here we designed a MA device that incorporates bull’s-eye features (Fig 4) to enable multiple significant advantages over traditional MA devices. Each bull’s-eye has an outer ring or groove (∼ 0.4 mm deep) called a ‘moat’ as well as a recess in the center of the bull’s-eye (same depth as moat). These simple macroscale features are introduced using the same soft-lithography technique commonly used to create the microwell devices themselves, thereby avoiding additional fabrication complexity. The moat passively leverages surface tension to isolate reagents and excess fluid into different regions of the array, enabling more than one experimental fluid to be applied to the same device for multiplexed experiments without cross-contamination. The moats also allow each bull’s-eye to be addressed using pipette transfers of up to 100 μL droplets instead of fluid overlays that span the entire device. Thus, they significantly reduce the finesse required for treatment and washing steps and thereby enable standardized fluid-handling steps for more repeatable results from user-to-user. The ability to perform MA fluid manipulations with a micro-pipette also enables potential use of standard liquid handling automation. The recess in the center of the region provides a site to place the pipette tip during aspiration and dispensation. The recess significantly reduces the chance of applying fluid shear directly over the microwells that could result in inadvertent aspiration or displacement of captured cells. Upon clamping a lid to the MA, excess fluid is displaced into the center recess and moat regions, again preventing cross contamination between bull’s-eyes. After placement of the lid, the excess fluid in the moat also acts to guard against potential evaporation through the gas/vapor permeable PDMS device for long-term kinetic studies. Although various MA designs exist, we are not aware of one that encompasses all of these capabilities on a single MA chip.

### Open-source software to streamline IDC

**Fig 5:**
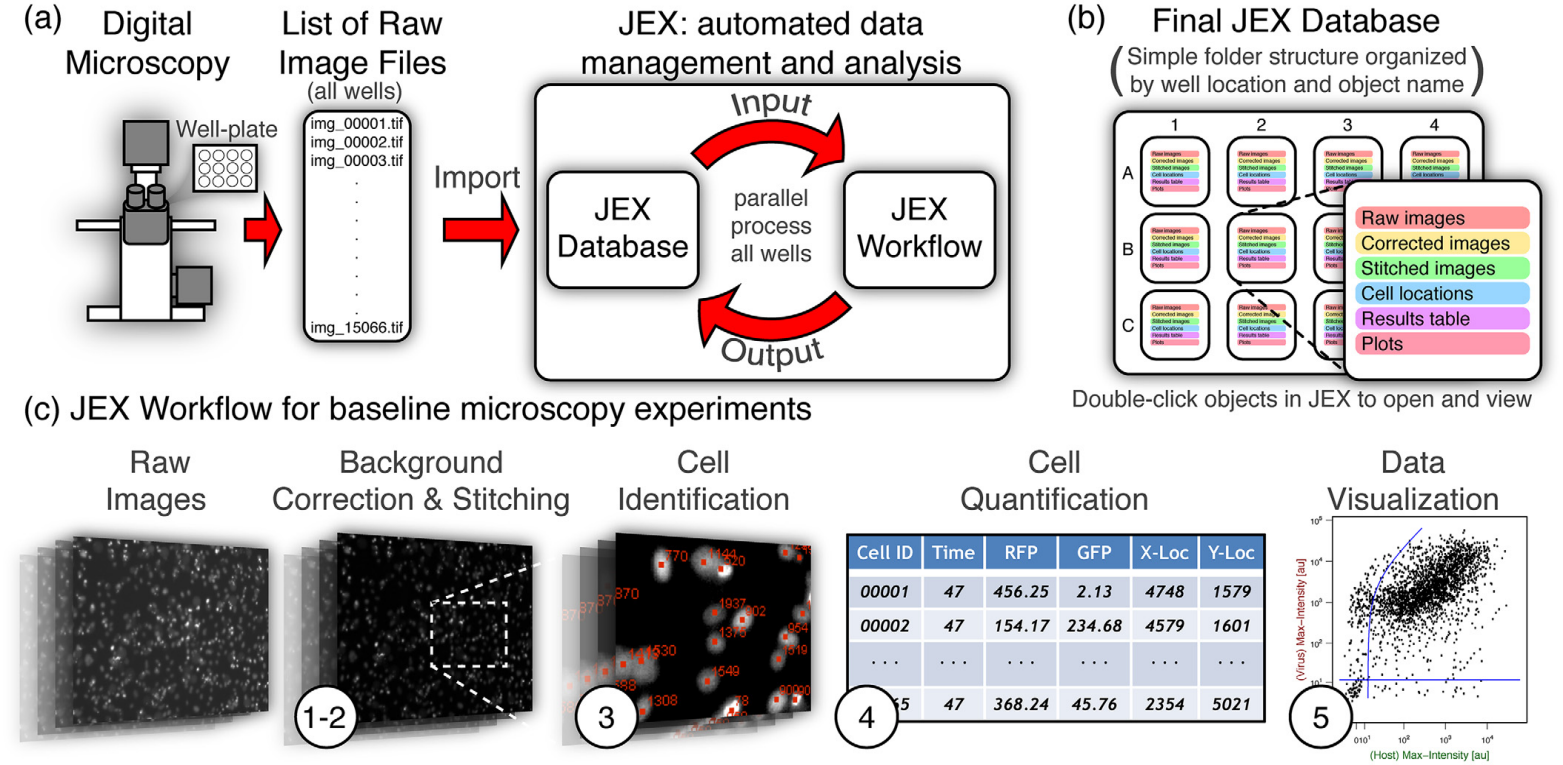
Data acquisition and analysis. a) Images are acquired in multiple colors and locations over time. The resultant list of files is imported into JEX. JEX performs analysis on the imported data, and stores the outputs into the same database. All function parameters and data from intermediate steps can be recorded in the JEX database. b) The database of information is stored in a simply named and transparent folder structure for perusal and use outside of JEX, but is also easily accessed via the JEX user interface. c) The workflow for baseline microscopy experiments generally consists of: 1) an initial background and illumination correction, 2) stitching the image array for each color and timepoint within each well, 3) identification of cells based on Hoechst staining, 4) quantification of cell location and fluorescence intensity for each color, and 5) plotting of results. The workflow for MA-based experiments includes additional functions for identifying microwells, counting the number of cells within each microwell, and quantifying whole-microwell and single-cell fluorescence. See S1 Text for details of all steps.

IDC image data was managed and processed using software called JEX [48] (Fig 5). JEX is a free, open-source Java application that provides a simple user interface for managing/databasing and processing large amounts of data, with specific focus on image data. Other notable related batch processing and databasing software packages include ImageJ/FIJI [49–51], CellProfiler [52], Knime [53], Icy [54], and OMERO [55]. Like these other software packages, a significant portion of the JEX image processing capability is enabled by the extensibility of ImageJ/ImageJ2/FIJI and the underlying libraries developed in parallel with them (*e.g.*, SciJava, ImgLib, BioFormats, and SCIFIO). The primary benefit of JEX, over these other more mature options is that databasing and batch processing are tightly integrated and given equal weight in the design and user interface of JEX. That is to say, JEX integrates batch processing and databasing into a single user interface while also visually organizing the database entries to mimic multiwell plates or other experimental setups for a simpler, more intuitive workflow. Thus, focus can be placed on analysis instead of data management to associate specific image sets with specific experimental conditions, helping to directly address logistical challenges associated with extensive microscopy experiments common to dynamic cytometry.

JEX also enables the use of R [56–58] and Octave [59, 60] (a free and open-source alternative to Matlab) from within JEX functions for integrating or automating downstream modeling and statistical analysis for further streamlining of protocols. This capability was used to automatically generate dynamic cytometry plots for rapid feedback on the population dynamics of each experiment (via the ‘Make Gated FACS Plots’ and ‘Make Movie’ functions).

The image processing algorithms used in this study are stored as JEX workflows. A JEX workflow is sequence of JEX functions with linked inputs and outputs and associated parameters. The functions used in each JEX workflow are included as part of the default function library in JEX. Furthermore, an example workflow, dataset, and instructions for quantifying single-cell fluorescence in microwells is included with the S1 Text and illustrates that JEX workflows can be saved and shared with those who have limited expertise in image processing. Details of image processing methodologies are also contained in the S1 Text.

### Validation Experiments

The primary components of the approach (*i.e.*, the biological system, MA-based device, and open-source software) were integrated and validated using three sets of experiments. The first two baseline experiments were performed in parallel and consisted of flow cytometry (FC) (referred to as FC experiments) and image analysis of infections in standard 12-well plate (referred to as microscopy experiments). The first two experiments establish baselines for validation of data obtained in the third set which employs the MAs to perform IDC (referred to as MA experiments).

A high-level summary of the validation experiments follows, while more detailed descriptions of the experimental protocol, image quantification, data fitting, and statistics are included in the S1 Text.

#### Baseline FC and microscopy experiments

Briefly, PC3-IFIT2 cells were cultured in 12-well plates on tissue culture polystyrene, infected with VSV, and quantified via FC and/or time-lapse microscopy. For time-lapse microscopy, images were acquired at 10X magnification on an incubated microscope. Acquisition of a 2 × 3 array of images every 10 minutes over the course of 15.5 hours in 9 wells for 3 fluorescent channels captured changing levels of protein expression using a total of 15066, 2048 × 2048 pixel, 16-bit images (i.e., ∼ 122 gigabytes of raw data). JEX was used to automate subsequent single-cell fluorescence analysis. In this case, the algorithm quantified fluorescence by identifying nuclei labeled with a live cell nuclear stain (Hoechst 33342) followed by quantification of the mean intensity within a small radius of the nucleus location (S1 Text). The signals from ∼ 1500 cells per condition/well produced a data set of roughly 3,766,500 data points that were tabulated and analyzed using the specialized data handling methods of JEX to maintain a low memory overhead for scalability.

Baseline FC and microscopy experiments were carried out in parallel. In order to obtain a time-course with standard FC, we fixed multiple samples at different times post infection (6, 12, and 18 hpi). Thus, each timepoint represents a different cell population. In contrast, each timecourse obtained with microscopy in a well-plate follows a single population and thus represents an approach to PDC. For a more accurate comparison, the 18 hpi timepoint in the microscopy experiments were immediately fixed and quantified via FC. Thus, the last timepoint in the FC data and the microscopy data of each matching condition represent the *same* population of cells quantified using both approaches.

#### MA-based IDC

PC3-IFIT2 cells were infected off-chip and seeded via pipette into the bull’s-eye regions of a MA device. A glass slide was clamped over the MA to isolate the confined cells. The entire bull’s eye (a 4-by-3 image array) was imaged every 30 min for > 24 hrs in 3 fluorescent channels at 4X magnification. JEX was used to pre-process timelapse images to correct for spatial and temporal variation in background intensity (S1 Text). Specialized JEX functions were also developed to automatically identify microwell locations, identify cells within microwells, and quantify whole-cell fluorescence from microwells containing a single cell. The quantification algorithm minimized signal cross-over between microwells while enabling exclusion of background (non-cell) pixels from intensity measurements for a ∼ 4-fold improvement in the signal-to-noise ratio compared to quantification of total microwell fluorescence (S1 Text).

A compressed file of fluorescence data from the baseline and MA experiments experiments is provided as supplementary information, S1 Data.

## Results and Discussion

### Baseline FC and microscopy experiments

**Fig 6:**
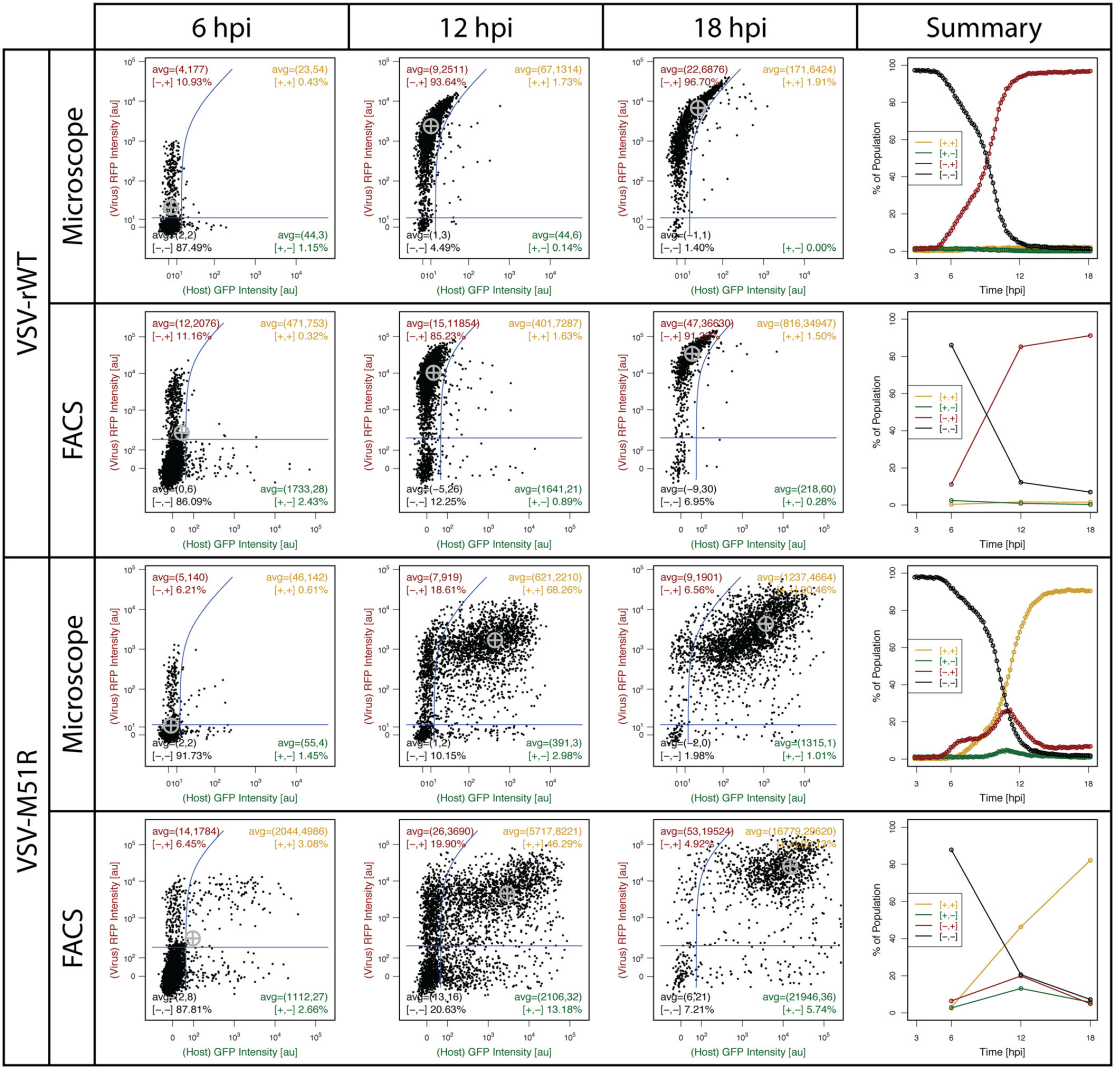
Movie frames of FC and microscopy PDC plots at 6, 12, and 18 hpi. The green and red fluorescence of each cell in VSV-rWT and VSV-M51R infections are shown in each scatter plot. All timepoints for the microscopy data are taken from the ***same*** sample whereas the FC data at each timepoint represents data from ***separate*** samples obtained in parallel. Furthermore, the microscope sample was sacrificed at 18 hpi and used for the FC 18 hpi timepoint. Thus, the 18 hpi timepoint is the same sample for both methods and is directly comparable. The summary plots on the right-hand side show gated population percentages over time, illustrating the similarity in sensitivity of the two methods but highlighting the improved temporal resolution of the microscopy approach. The gray cross-hair in each plot represents the overall population mean.

FC and microscopy results at 6, 12, and 18 hpi are shown in Fig 6 for comparison. Within the scatter plots, the threshold values (3*σ*) are estimated based on basal expression levels at the first timepoint (i.e., prior to infection progression, see S1 Text for associated plots). Unless otherwise noted throughout, the default ‘mad’ function (median absolute deviation) in the R software is used as an estimator of the standard deviation, *σ*, due to its robustness to outliers. The blue threshold lines exhibit curvature on the log-scale fluorescence plots to account for a small constant fraction of spectral overlap or crossover of signal from the red fluorescence channel to the green that was present in both microscope and flow cytometry instrumentation. Cross-over the other direction was negligible. The axes of the plots are on a ‘logicle’ scale which transitions from a linear scale to logarithmic at the blue threshold lines to enable plotting of a wide dynamic range while accurately representing the background noise of values near 0. The gray ‘cross-hair’ represents the population mean. Each scatter plot also shows gated population percentages and intensity means for cells in each ‘quadrant’. The last column of Fig 6 plots the gated percentages for each imaged timepoint.

Given the temporal resolution of the microscopy data, we leveraged JEX and its connection to R to compile the microscopy plots into image stacks and render them as movies, termed PDC plots. The PDC plots provide a powerful means of representing the dynamic behavior of individual cells and sub-populations. PDC plots/movies are provided in S2–S4 Movies along with an example timelapse movie of microscopy data for VSV-M51R (S1 Movie). One general phenotype evident in the timelapse movie is the rounded morphology of many of the cells. It is well established that expression of the matrix (M) protein of VSV causes cell rounding, likely by depolymerization of microtubules.[61] This cytopathic effect is apparent not only in the cells in baseline experiments but also MA experiments, (S5 Movie).

Data in Fig 6 demonstrate a similar detection sensitivity (i.e., time of detection) and dynamic range (i.e., the range from the calculated 3*σ* threshold to the highest detected cellular fluorescence), as well as percentages of gated populations over time, generally validating the microscopy-based method of quantification for flow-cytometry-like readouts. As expected, the VSV-rWT infections produce ample viral protein (RFP^+^) and elicited almost no host activation (GFP^−^) while the large majority of VSV-M51R infections (∼ 90% of cells or more) produced high levels of both viral protein and host activation (RFP^+^GFP^+^).

Beyond validation, the data also demonstrate the extensive heterogeneity in virus-host interactions. The location of the population average at each time point (gray cross-hair) in most cases represents the behavior of only a small fraction of the total cell population and highlights the vast heterogeneity of behaviors that is missed by using average population measures. The movie version of the PDC plots contained in the S1 Text illustrate the kinetic heterogeneity of virus-host interactions. It appears that in most cells the viral reporter rises first, while in a minority of cells the host response reporter can be readily detected before the viral reporter. In this way, cells appear to take different ‘trajectories’ through the plot of red *vs* green, passing through and remaining in the various quadrants for different amounts of time and in different sequence. The approach also provides physical location information for each cell (*i.e.*, x-y pixel coordinates) that could be used to leverage spatial heterogeneity as done by Snijder *et al*. or in cell profiling applications which leverage spatial context during single-cell characterization [8, 62]. It is also imagined the information can be used to analyze virus-host interactions in spatially spreading infections [31].

In summary, these baseline experiments validate the quantitative capabilities and scalability of our microscopy approach and JEX for dynamic cytometry. They also highlight the extensive heterogeneity of individual cell trajectories in their timing and level of expression of viral and host response reporters as well as the future potential for spatial analysis of spreading infections. We now add MAs for cell-confinement to advance from PDC to IDC, using these baseline results for comparison and validation.

### MA-based IDC

MAs have been used in myriad applications including single-cell PCR [43, 44, 63], protein secretion [64], cell-cell interactions [40], and IDC [41, 65]. The study performed by Roach *et al.* is one of the earliest demonstrations of how MAs enable unambiguous tracking and quantification of single-cell intracellular fluorescence kinetics using a single fluorescent readout; however, others have also recognized and highlighted this potential [41, 66]. Furthermore, the considerable work done by the Love group and others to quantify cell secretion and physical cell-cell interactions over time are notable demonstrations of different types of IDC endpoints [40, 64].

The MA-based approach to IDC presented here is unique in multiple respects. The novel bull’s-eye MA design enables pipette-based operation and simplified treatment, washing, sealing, and isolation of separate treatment conditions on a single chip. The biological application is also unique, focusing on enabling detailed kinetics of competing virus-host interactions using a unique engineered dual-color virus-host system. We have also developed and integrated our approach with an open-source software package that specifically addresses the issue of data management for IDC applications while enabling streamlined and flexible automation of data analysis challenges specific to MA-based assays.

**Fig 7:**
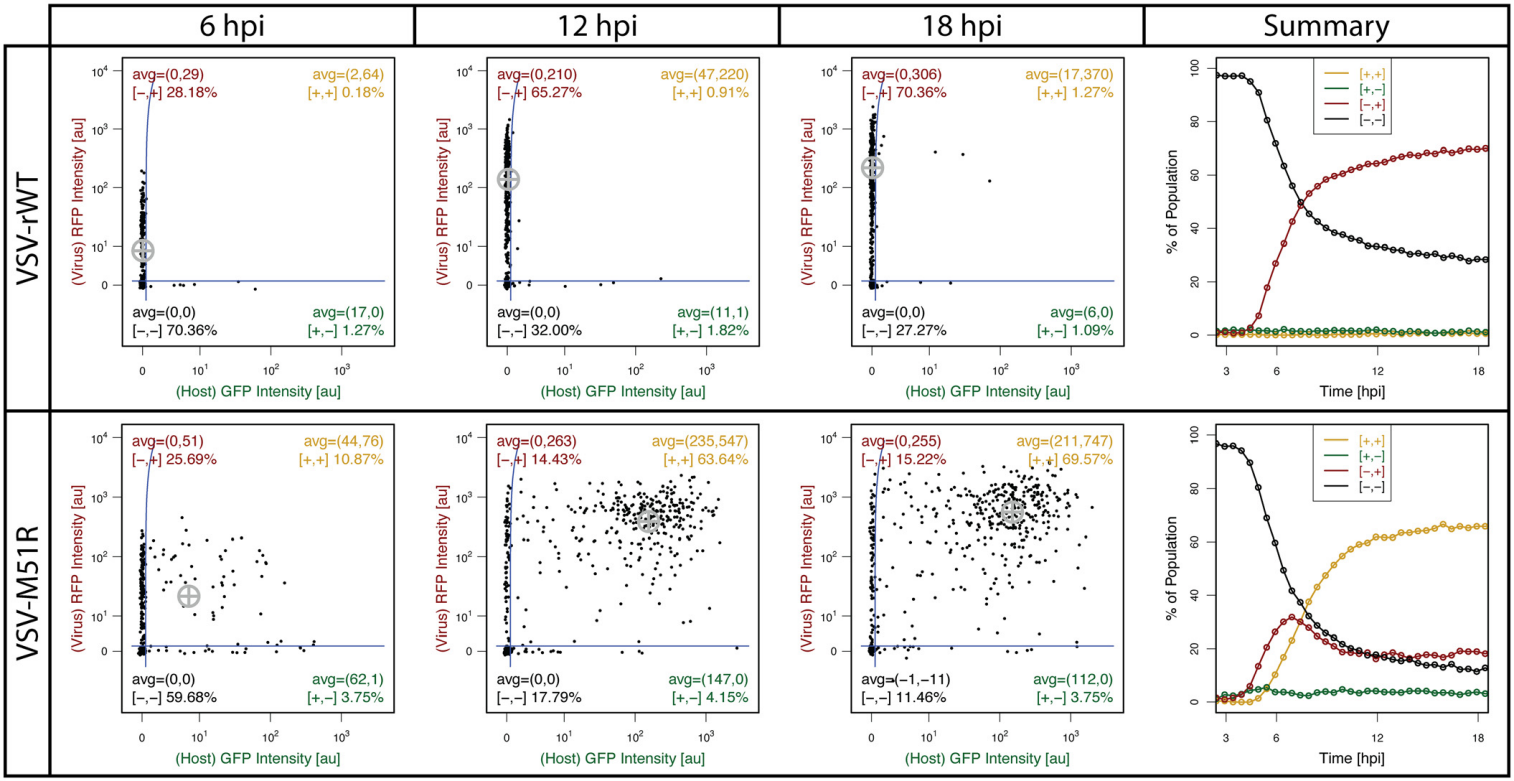
Microwell-array-based cytometry plots (VSV-M51R & VSV-rWT) show agreement with results from baseline cytometry experiments from Fig 6.

Data collected using this MA-based approach to IDC is compared to the baseline experiments. Individual timepoints of the PDC plots and summary plots of VSV-M51R infections reveal similar trends and some differences between MA and baseline results (Figs 6 and 7, S6 Movie). The summary plot for each dataset shows similar distributions of cellular fluorescence intensity at 6, 12, and 18 hpi as well as similar trends in the frequency of the 4 gated populations. However, the percentage of GFP^−^/RFP^−^cells falls more quickly and GFP^−^/RFP^+^ cells are initially detectable ∼ 1 hours earlier for cells in MAs than in baseline experiments. These differences may reflect, in part, differences in the initiations of infection (see S1 Text). Briefly, cells in MA experiments were treated with virus in suspension in a tube on ice to promote adsorption without entry [19, 67], then washed three times in the cold to remove free particles without entry, incubated at 37°C for 7 min to allow viral entry, and immediately transferred to the MA for imaging. This was done to minimize time between infection and imaging, which is separated by 20–30 min for seeding and sealing the MA device and microscope setup. By contrast, virus for baseline experiments was incubated with cells for 20 minutes at 37°C, followed by washing with media and placement on the microscope for imaging. Thus, virus in MA experiments was ‘pre-adsorbed’ and provided a shorter window of time for entry prior to wash (7 min vs 20 min), likely producing a more synchronized start to infection that allows for earlier detection in the summary plot. The baseline results also exhibit a ‘two-stage’ rise in the number of infected cells compared to MA results. Baseline experiments allow successfully infected cells to spread to adjacent cells that may not have been successfully infected initially, producing a second round of infection. In MA experiments, microwells prevent spread of virus well-to-well and analysis is limited to singly isolated cells; therefore, there is no opportunity for multiple rounds of infection to be observed. The physical isolation of infections is an fundamental difference between the methodologies and might also play a role in the observed difference in final values of the GFP^−^RFP^+^ population between baseline and MA experiments (18% vs 7%) and the corresponding reduction of the GFP^+^RFP^+^ cell population. The microwells isolate and deprive each cell of antiviral cytokines from neighboring cells, potentially lowering the frequency of activated cells compared to a population context of the baseline experiments where soluble factor communication can exist between cells. Thus, the physical isolation enabled by MAs should be considered when interpreting results using this approach, but also provides a potential opportunity to distinguish between effects of autocrine versus paracrine signaling depending on microwell device setup [27].

The MA-based approach also addressed important challenges specific to studying virus infections. Given the significant cell density, number of timepoints, and amount of pathology, lysis, and migration in the infected cells, cell tracking on a standard 2D culture substrate using nearest neighbor methods, more advanced linear assignment methods (with and without manual curation)[22], as well as completely manual tracking were unproductive. Manual curation (*i.e.*, elimination or fixing of erroneous tracks) did not scale well for datasets beyond ∼ 100 tracks. Success was highly dependent on the time between images given cells were moving quickly in a dense population. Completely automated methods appeared to track less mobile cells more robustly, thus potentially biasing analysis. Physically isolating individual cells in microwells eliminated the need for tracking algorithms and enabled detailed whole-cell kinetic studies amidst such challenges. This method also posed less risk of aerosolized virus compared to FC, enabling dynamic study and re-interrogation of live cells infected with competent virus. The new MA embodiment also allowed us to perform mock and infected controls on the same chip instead of requiring separate devices. This allowed us to avoid potential device-to-device variability and microscope/incubation variability from day-to-day.

**Fig 8:**
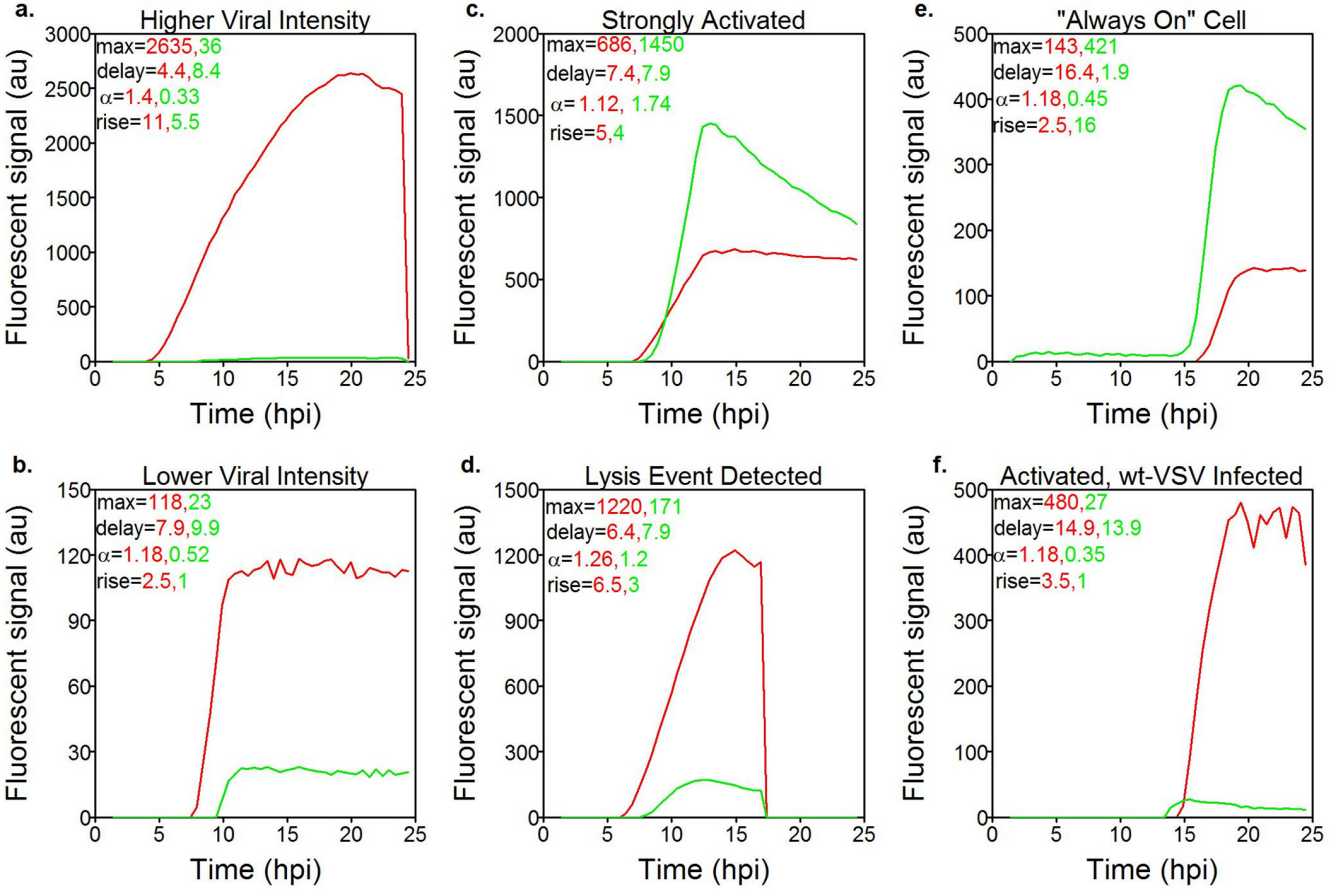
Sample VSV-rWT viral and innate immune reporter protein expression kinetics from six individual cells. Kinetic parameters have been extracted from the trajectories of both the viral (red) and host (green) trajectories and displayed in the top left corner of each figure. Maximum yields from individual cells vary greatly (a-b), host reporter expression can be greater or lesser than viral gene expression given similar starting conditions (c-d), and cells can lyse (d) or remain intact during imaging. Single-cell analysis can detect and quantify cells displaying rare behavior, such as those that appear to have a basal level of innate immune activation all the time (e) and cells infected with wt-VSV that express ZS-Green despite encoding a functional matrix gene.

The approach also enables high-fidelity analysis of kinetics. The intensity of each cell in each fluorescence channel through time is termed a trajectory. Fig 8 illustrates qualitatively some different ‘types’ of trajectories or behaviors present in the data. The examples illustrate many different ways in which infections can progress, and include a wide range of overall reporter protein yields. Virus and host fluorescence trajectories were also used to estimate characteristic kinetic parameters including max intensity (max), time-delay to fluorescent protein production (delay), the initial exponential growth rate upon production (*α*), and the time between initial production and plateau of production (rise). Lastly, if cell lysis is detected, the time of lysis is recorded. See the S1 Text for details on quantification of these metrics. This enables quantitative multi-parametric kinetic analysis of competing viral and host processes. Although the data are from just a single set of paired experiments, these data demonstrate an integrated method and approach for detailed interrogation of virus-host interaction heterogeneity and dynamics.

The fidelity of the approach enabled us to be confident in our observations of rare events in the MA experiments such as VSV-rWT infected cells that become activated (Fig 8f, S6 Movie). These rare events are corroborated by similar observations in baseline experiments (Figs 6 and 7, S7 Movie). Likewise, it can also be seen that the innate immune response, as measured by IFIT2 reporter expression, is typically inactive initially, yet some cells are active well before detection of infection progression (Fig 8e). Indeed single cell variability observed in baseline experiments of non-infected cells suggest some cells might be active prior to infection (Fig 2, S1 Text). Isolation of each infection enabled detection of cell lysis, which occurred in a significant fraction of infections; however, detailed analyses of correlations between such events and parameters of infections are outside the scope of this discussion and left for follow-on studies.

The MA-based method also improves throughput for high-fidelity quantification of single-cell kinetics (IDC). The MA device has ∼ 2500 microwells per bull’s eye, of which 340 ± 60 (mean ± sd) contained only a single cell. A total of 8 bull’s eyes could be imaged at once, thus providing an opportunity to unambiguously track 2712 individual cells throughout the experiment for kinetic analysis. Although data from all these cells could be analyzed, analysis was limited to the 39% of cells where there was detectable RFP for a subtotal of 1056 (505 VSV-M51R and 550 VSV-rWT). In comparison, advance cell tracking applied to the 2900 ± 500 cells imaged per well in the microscopy experiment was likely to result in ∼ 15–20% of cell tracks being invalid [28–30]. Therefore, we did not venture to manually curate the data, which would still not guarantee fidelity, but instead performed PDC analysis, calculating population characteristics at each time point. FC experiments gathered ∼ 10,000 datapoints per condition and provided the highest throughput, but required parallel samples to perform PDC. Lastly, in previous work, limiting dilution was used to isolate single cells in a standard 24-well plate, of which 12 were successfully infected and unambiguously tracked for single-cell kinetics analysis of virus production (IDC).[19] Thus, although the raw throughput (i.e., number of cells studied) of MAs is well-below flow cytometry and standard microscopy, the throughput for unambiguous, high-fidelity, single-cell kinetics analysis (IDC) is significantly improved.

## Conclusion

Methods for individual-cell dynamic cytometry (IDC) are able to address unique sets of questions compared to static cytometry or PDC methods which are unable to follow single-cells through time. These methods are poised and already making significant contributions to a broad range of topics where dynamics and or cellular heterogeneity play important roles such as development, cancer, immunology, and virology. However, these approaches are often costly, complex, and/or challenging across multiple aspects from readout development and imaging to analysis and interpretation. Thus, advances to streamline IDC are needed to achieve broader adoption for accelerated progress. To help address this need we have advanced an integrated set of tools consisting of: 1) a dual-color virus-host system with matched fluorescence kinetics; 2) a new MA-based device embodiment that streamlines protocols for single-cell isolation, tracking, and quantification; and 3) a set of batch processing algorithms for microwell analysis within a free and open-source software package called JEX for image analysis and databasing. We validated these integrated components for single-cell fluorescence quantification against flow-cytometry and microscopy of standard 2D cultures. The MA-based approach provides high-fidelity time-course data that enables parametric quantification of viral infection kinetics that has already been used to elucidate co-infections of virus with defective interfering virus-like particles [68], and will be leveraged to probe the biology of virus and immune response dynamics. This study provides a base to advance IDC methods for new insights that cannot be obtained with other static or population-based approaches.

## Supporting Information

S1 Text Supporting Information. Topics include cell culture; VSV RFP *vs* plaque forming units; background subtraction and illumination correction; Baseline experiment cell preparation, data acquisition, analysis, and supplemental PDC plots; microwell device fabrication, preparation, seeding, and clamping; automated microwell identification; quantification of whole-cell intensity; analysis in R; curve fitting of fluorescence kinetics; quantification of cell lysis; PDC plots of control conditions; and instructions for microwell analysis using JEX on a provided example dataset.
(PDF)

S1 Movie Fluorescence timelapse movie of VSV-M51R condition in standard 2D culture baseline experiment. (blue) Hoechst 33342 nuclear stain, (red) viral DsRed-Express DR reporter protein, and (green) host IFIT2 Zs-Green reporter protein.
(MOV)

S2 Movie Baseline PDC plot of VSV-rWT condition in standard 2D culture baseline experiments. (MP4)

S3 Movie PDC plot of VSV-M51R condition in standard 2D culture baseline experiments. (MP4)

S4 Movie Baseline PDC plot of mock condition in standard 2D culture baseline experiments. (MP4)

S5 Movie Fluorescence timelapse movie of VSV-M51R condition in MA experiment. (blue) Hoechst 33342 nuclear stain, (red) viral DsRed-Express DR reporter protein, and (green) host IFIT2 Zs-Green reporter protein.
(MOV)

S6 Movie PDC plot of VSV-rWT condition in MA experiments. (MP4)

S7 Movie PDC plot of VSV-M51R condition in MA experiments. (MP4)

S1 Data Compressed file of fluorescence data for baseline and MA experiments. (ZIP)

## References

[1] Marjanovic ND, Weinberg RA, Chaffer CL. Cell plasticity and heterogeneity in cancer. Clin Chem. 2013 1;59(1):168–79. 10.1373/clinchem.2012.184655 23220226 PMC6220421

[2] Meacham CE, Morrison SJ. Tumour heterogeneity and cancer cell plasticity. Nature. 2013 9;501(7467):328–37. 10.1038/nature12624 24048065 PMC4521623

[3] Rand U, Rinas M, Schwerk J, Nöhren G, Linnes M, Kröger A, et al Multi-layered stochasticity and paracrine signal propagation shape the type-I interferon response. Mol Syst Biol. 2012;8:584 10.1038/msb.2012.17 22617958 PMC3377992

[4] Tonegawa S. Somatic generation of antibody diversity. Nature. 1983 4;302(5909):575–81. 10.1038/302575a0 6300689

[5] Abbas AK, Murphy KM, Sher A. Functional diversity of helper T lymphocytes. Nature. 1996 10;383(6603):787–93. 10.1038/383787a0 8893001

[6] Huang S. Non-genetic heterogeneity of cells in development: more than just noise. Development. 2009 12;136(23):3853–62. 10.1242/dev.035139 19906852 PMC2778736

[7] Zernicka-Goetz M, Huang S. Stochasticity versus determinism in development: a false dichotomy? Nat Rev Genet. 2010 11;11(11):743–4. 20877326 10.1038/nrg2886

[8] Snijder B, Sacher R, Rämö P, Damm EM, Liberali P, Pelkmans L. Population context determines cell-to-cell variability in endocytosis and virus infection. Nature. 2009 9;461(7263):520–3. 10.1038/nature08282 19710653

[9] Muzzey D, van Oudenaarden A. Quantitative time-lapse fluorescence microscopy in single cells. Annu Rev Cell Dev Biol. 2009;25:301–27. 10.1146/annurev.cellbio.042308.113408 19575655 PMC3137897

[10] Pelkmans L. Cell Biology. Using cell-to-cell variability—a new era in molecular biology. Science. 2012 4;336(6080):425–6. 10.1126/science.1222161 22539709

[11] Sisan DR, Halter M, Hubbard JB, Plant AL. Predicting rates of cell state change caused by stochastic fluctuations using a data-driven landscape model. Proc Natl Acad Sci U S A. 2012 11;109(47):19262–7. 10.1073/pnas.1207544109 23115330 PMC3511108

[12] Patil S, Fribourg M, Ge Y, Batish M, Tyagi S, Hayot F, et al Single-cell analysis shows that paracrine signaling by first responder cells shapes the interferon-*β* response to viral infection. Sci Signal. 2015 2;8(363):ra16 10.1126/scisignal.2005728 25670204

[13] Rand U, Hillebrand U, Sievers S, Willenberg S, Köster M, Hauser H, et al Uncoupling of the dynamics of host-pathogen interaction uncovers new mechanisms of viral interferon antagonism at the single-cell level. Nucleic Acids Res. 2014 7;42(13):e109 10.1093/nar/gku492 24895433 PMC4117750

[14] Shalek AK, Satija R, Shuga J, Trombetta JJ, Gennert D, Lu D, et al Single-cell RNA-seq reveals dynamic paracrine control of cellular variation. Nature. 2014 6;510(7505):363–9. 10.1038/nature13437 24919153 PMC4193940

[15] Zhao M, Zhang J, Phatnani H, Scheu S, Maniatis T. Stochastic expression of the interferon-*β* gene. PLoS Biol. 2012 1;10(1):e1001249 10.1371/journal.pbio.1001249 22291574 PMC3265471

[16] Hensel SC, Rawlings JB, Yin J. Stochastic kinetic modeling of vesicular stomatitis virus intracellular growth. Bull Math Biol. 2009 10;71(7):1671–92. 10.1007/s11538-009-9419-5 19459014 PMC3169382

[17] Srivastava R, You L, Summers J, Yin J. Stochastic vs. deterministic modeling of intracellular viral kinetics. J Theor Biol. 2002 10;218(3):309–21. 10.1006/jtbi.2002.3078 12381432

[18] Zhu Y, Yongky A, Yin J. Growth of an RNA virus in single cells reveals a broad fitness distribution. Virology. 2009 3;385(1):39–46. 10.1016/j.virol.2008.10.031 19070881 PMC2666790

[19] Timm A, Yin J. Kinetics of virus production from single cells. Virology. 2012 3;424(1):11–7. 10.1016/j.virol.2011.12.005 22222212 PMC3268887

[20] Castellarnau M, Szeto GL, Su HW, Tokatlian T, Love JC, Irvine DJ, et al Stochastic particle barcoding for single-cell tracking and multiparametric analysis. Small. 2015 1;11(4):489–98. 10.1002/smll.201401369 25180800 PMC4303509

[21] Di Carlo D, Wu LY, Lee LP. Dynamic single cell culture array. Lab Chip. 2006 11;6(11):1445–9. 10.1039/b605937f 17066168

[22] Jaqaman K, Loerke D, Mettlen M, Kuwata H, Grinstein S, Schmid SL, et al Robust single-particle tracking in live-cell time-lapse sequences. Nat Methods. 2008 8;5(8):695–702. 10.1038/nmeth.1237 18641657 PMC2747604

[23] Kretzschmar K, Watt FM. Lineage tracing. Cell. 2012 1;148(1-2):33–45. 10.1016/j.cell.2012.01.002 22265400

[24] Mali P, Aach J, Lee JH, Levner D, Nip L, Church GM. Barcoding cells using cell-surface programmable DNA-binding domains. Nat Methods. 2013 5;10(5):403–6. 10.1038/nmeth.2407 23503053 PMC3641172

[25] Valero A, Post JN, van Nieuwkasteele JW, Ter Braak PM, Kruijer W, van den Berg A. Gene transfer and protein dynamics in stem cells using single cell electroporation in a microfluidic device. Lab Chip. 2008 1;8(1):62–7. 10.1039/B713420G 18094762

[26] Wheeler AR, Throndset WR, Whelan RJ, Leach AM, Zare RN, Liao YH, et al Microfluidic device for single-cell analysis. Anal Chem. 2003 7;75(14):3581–6. 10.1021/ac0340758 14570213

[27] Yamanaka YJ, Szeto GL, Gierahn TM, Forcier TL, Benedict KF, Brefo MSN, et al Cellular barcodes for efficiently profiling single-cell secretory responses by microengraving. Anal Chem. 2012 12;84(24):10531–6. 10.1021/ac302264q 23205933 PMC3691955

[28] Maška M, Ulman V, Svoboda D, Matula P, Matula P, Ederra C, et al A benchmark for comparison of cell tracking algorithms. Bioinformatics. 2014 6;30(11):1609–17. 10.1093/bioinformatics/btu080 24526711 PMC4029039

[29] Kan A, Chakravorty R, Bailey J, Leckie C, Markham J, Dowling MR. Automated and semi-automated cell tracking: addressing portability challenges. J Microsc. 2011 11;244(2):194–213. 10.1111/j.1365-2818.2011.03529.x 21895653

[30] Li K, Miller ED, Chen M, Kanade T, Weiss LE, Campbell PG. Cell population tracking and lineage construction with spatiotemporal context. Med Image Anal. 2008 10;12(5):546–66. 10.1016/j.media.2008.06.001 18656418 PMC2670445

[31] Swick A, Baltes A, Yin J. Visualizing infection spread: dual-color fluorescent reporting of virus-host interactions. Biotechnol Bioeng. 2014 6;111(6):1200–9. 10.1002/bit.25170 24338628 PMC4004699

[32] Fensterl V, Wetzel JL, Ramachandran S, Ogino T, Stohlman SA, Bergmann CC, et al Interferon-induced Ifit2/ISG54 protects mice from lethal VSV neuropathogenesis. PLoS Pathog. 2012;8(5):e1002712 10.1371/journal.ppat.1002712 22615570 PMC3355090

[33] Müller U, Steinhoff U, Reis LF, Hemmi S, Pavlovic J, Zinkernagel RM, et al Functional role of type I and type II interferons in antiviral defense. Science. 1994 6;264(5167):1918–21. 10.1126/science.8009221 8009221

[34] Ahmed M, Lyles DS. Identification of a consensus mutation in M protein of vesicular stomatitis virus from persistently infected cells that affects inhibition of host-directed gene expression. Virology. 1997 10;237(2):378–88. 10.1006/viro.1997.8808 9356348

[35] Lyles DS, Rupprecht CE. Fields Virology: Rhabdoviridae. pp 1363–1408, 5th ed Knipe DM, Howley P, editors. Philadelphia, PA: Lippincott Williams & Wilkins; 2007.

[36] Barr JN, Whelan SP, Wertz GW. Role of the intergenic dinucleotide in vesicular stomatitis virus RNA transcription. J Virol. 1997 3;71(3):1794–801. 9032308 10.1128/jvi.71.3.1794-1801.1997 PMC191248

[37] Flanagan EB, Zamparo JM, Ball LA, Rodriguez LL, Wertz GW. Rearrangement of the genes of vesicular stomatitis virus eliminates clinical disease in the natural host: new strategy for vaccine development. J Virol. 2001 7;75(13):6107–14. 10.1128/JVI.75.13.6107-6114.2001 11390612 PMC114326

[38] Sen GC, Sarkar SN. The interferon-stimulated genes: targets of direct signaling by interferons, double-stranded RNA, and viruses. Curr Top Microbiol Immunol. 2007;316:233–50. 17969451 10.1007/978-3-540-71329-6_12

[39] Carey BL, Ahmed M, Puckett S, Lyles DS. Early steps of the virus replication cycle are inhibited in prostate cancer cells resistant to oncolytic vesicular stomatitis virus. J Virol. 2008 12;82(24):12104–15. 10.1128/JVI.01508-08 18829743 PMC2593309

[40] Yamanaka YJ, Berger CT, Sips M, Cheney PC, Alter G, Love JC. Single-cell analysis of the dynamics and functional outcomes of interactions between human natural killer cells and target cells. Integr Biol (Camb). 2012 10;4(10):1175–84. 10.1039/c2ib20167d 22945136

[41] Roach KL, King KR, Uygun BE, Kohane IS, Yarmush ML, Toner M. High throughput single cell bioinformatics. Biotechnol Prog. 2009;25(6):1772–9. 10.1002/btpr.289 19830811 PMC3208255

[42] Ozawa T, Kinoshita K, Kadowaki S, Tajiri K, Kondo S, Honda R, et al MAC-CCD system: a novel lymphocyte microwell-array chip system equipped with CCD scanner to generate human monoclonal antibodies against influenza virus. Lab Chip. 2009 1;9(1):158–63. 10.1039/B810438G 19209349

[43] Nagai H, Murakami Y, Morita Y, Yokoyama K, Tamiya E. Development of a microchamber array for picoliter PCR. Anal Chem. 2001 3;73(5):1043–7. 10.1021/ac000648u 11289415

[44] Gong Y, Ogunniyi AO, Love JC. Massively parallel detection of gene expression in single cells using subnanolitre wells. Lab Chip. 2010 9;10(18):2334–7. 10.1039/c004847j 20686711 PMC4040084

[45] Choi J, Love KR, Gong Y, Gierahn TM, Love JC. Immuno-hybridization chain reaction for enhancing detection of individual cytokine-secreting human peripheral mononuclear cells. Anal Chem. 2011 9;83(17):6890–5. 10.1021/ac2013916 21812465 PMC3175753

[46] Ogunniyi AO, Story CM, Papa E, Guillen E, Love JC. Screening individual hybridomas by microengraving to discover monoclonal antibodies. Nat Protoc. 2009;4(5):767–82. 10.1038/nprot.2009.40 19528952 PMC4034573

[47] Love KR, Bagh S, Choi J, Love JC. Microtools for single-cell analysis in biopharmaceutical development and manufacturing. Trends Biotechnol. 2013 5;31(5):280–6. 10.1016/j.tibtech.2013.03.001 23582471

[48] Warrick JW, Berthier E. Je’Xperiment (JEX);. Available from: https://github.com/jaywarrick/JEX.

[49] Schneider CA, Rasband WS, Eliceiri KW. NIH Image to ImageJ: 25 years of image analysis. Nat Methods. 2012 7;9(7):671–5. 10.1038/nmeth.2089 22930834 PMC5554542

[50] Rueden C, Schindelin J, Hiner M, DeZonia B, Kamentsky L, Eliceiri K. ImageJ2;. Available from: http://imagej.net. 10.1186/s12859-017-1934-z PMC5708080 29187165

[51] Schindelin J, Arganda-Carreras I, Frise E, Kaynig V, Longair M, Pietzsch T, et al Fiji: an open-source platform for biological-image analysis. Nat Methods. 2012 7;9(7):676–82. 10.1038/nmeth.2019 22743772 PMC3855844

[52] Carpenter AE, Jones TR, Lamprecht MR, Clarke C, Kang IH, Friman O, et al CellProfiler: image analysis software for identifying and quantifying cell phenotypes. Genome Biol. 2006;7(10):R100 10.1186/gb-2006-7-10-r100 17076895 PMC1794559

[53] Berthold MR, Cebron N, Dill F, Gabriel TR, Kötter T, Meinl T, et al KNIME: The Konstanz Information Miner In: Studies in Classification, Data Analysis, and Knowledge Organization (GfKL 2007). Springer; 2007 Available from: http://tech.knime.org/community/image-processing.

[54] de Chaumont F, Dallongeville S, Chenouard N, Hervé N, Pop S, Provoost T, et al Icy: an open bioimage informatics platform for extended reproducible research. Nat Methods. 2012 7;9(7):690–6. 10.1038/nmeth.2075 22743774

[55] Allan C, Burel JM, Moore J, Blackburn C, Linkert M, Loynton S, et al OMERO: flexible, model-driven data management for experimental biology. Nat Methods. 2012 3;9(3):245–53. 10.1038/nmeth.1896 22373911 PMC3437820

[56] R Core Team. R: A Language and Environment for Statistical Computing. Vienna, Austria; 2014 Available from: http://www.R-project.org/.

[57] Hornik K, Leisch F, Zeileis A, editors. Rserve: A Fast Way to Provide R Functionality to Applications; 2003 ISSN 1609–395X.

[58] Urbanek S. rJava: Low-Level R to Java Interface;. Available from: http://www.rforge.net/rJava/.

[59] Eaton JW, Bateman D, Hauberg S. GNU Octave version 3.0.1 manual: a high-level interactive language for numerical computations CreateSpace Independent Publishing Platform; 2009 ISBN 1441413006. Available from: http://www.gnu.org/software/octave/doc/interpreter.

[60] Hansen K. JavaOctave;. Available from: https://kenai.com/projects/javaoctave/pages/Home.

[61] Lyles DS, McKenzie MO. Activity of vesicular stomatitis virus M protein mutants in cell rounding is correlated with the ability to inhibit host gene expression and is not correlated with virus assembly function. Virology. 1997 3;229(1):77–89. 10.1006/viro.1996.8415 9123880

[62] Jones TR, Carpenter AE, Lamprecht MR, Moffat J, Silver SJ, Grenier JK, et al Scoring diverse cellular morphologies in image-based screens with iterative feedback and machine learning. Proc Natl Acad Sci U S A. 2009 2;106(6):1826–31. 10.1073/pnas.0808843106 19188593 PMC2634799

[63] Lindström S, Hammond M, Brismar H, Andersson-Svahn H, Ahmadian A. PCR amplification and genetic analysis in a microwell cell culturing chip. Lab Chip. 2009 12;9(24):3465–71. 10.1039/b912596e 20024024

[64] Love JC, Ronan JL, Grotenbreg GM, van der Veen AG, Ploegh HL. A microengraving method for rapid selection of single cells producing antigen-specific antibodies. Nat Biotechnol. 2006 6;24(6):703–7. 10.1038/nbt1210 16699501

[65] Haselgrübler T, Haider M, Ji B, Juhasz K, Sonnleitner A, Balogi Z, et al High-throughput, multiparameter analysis of single cells. Anal Bioanal Chem. 2013 12;. 24292433 10.1007/s00216-013-7485-x

[66] Rettig JR, Folch A. Large-scale single-cell trapping and imaging using microwell arrays. Anal Chem. 2005 9;77(17):5628–34. 10.1021/ac0505977 16131075

[67] Matlin KS, Reggio H, Helenius A, Simons K. Pathway of vesicular stomatitis virus entry leading to infection. J Mol Biol. 1982 4;156(3):609–31. 10.1016/0022-2836(82)90269-8 6288961

[68] Akpinar F, Timm A, Yin J. High-throughput single-cell kinetics of virus infections in the presence of defective interfering particles. Journal of Virology. 2015;(accepted). 10.1128/JVI.02190-15 26608322 PMC4719634

