# Supplementary material for: Tools for Single-Cell Kinetic Analysis of Virus-Host Interactions
Source: PLoS One. 2016 Jan 11;11(1):e0145081. doi: 10.1371/journal.pone.0145081 (PMC4713429; doi:10.1371/journal.pone.0145081)
Supplement: S1 Text — Topics include cell culture; VSV RFP vs plaque forming units; background subtraction and illumination correction; Baseline experiment cell preparation, data acquisition, analysis, and supplemental PDC plots; microwell device fabrication, preparation, seeding, and clamping; automated microwell identification; quantification of whole-cell intensity; analysis in R; curve fitting of fluorescence kinetics; quantification of cell lysis; PDC plots of control conditions; and instructions for microwell analysis using JEX on a provided example dataset. (PDF) [file pone.0145081.s001.pdf]

S1 Text. Supporting Information

1 General Methods and Information

1.1 Cell culture

PC3-IFIT2 reporter cells were maintained in RPMI 1640 10% FBS at 37°C 5%CO_2 and subcultured twice per week. Reporter cells were kept at low passage post monoclonal selection (10-12 passages). Prior to infection cells were harvested at 90% confluency by trypsinization, counted by hemocytometer and diluted to approximately 3.5x10^5 cells/ml. Cells were plated at that density in a multi-well tissue culture plate, allowed to sit ∼ 15 minutes at RT to promote a more uniform monolayer, and incubated for at least 12 hrs at 37°C 5%CO_2 prior to treatment or infection.

All work was done in a BSL2 laboratory setting.

1.2 VSV RFP *vs* PFU

The relationship of viral RFP production to the resultant number of plaque forming units (PFU) for the entire population was examined and is plotted in Fig. A. The data shows that RFP appears to be a good surrogate for virus particle production.

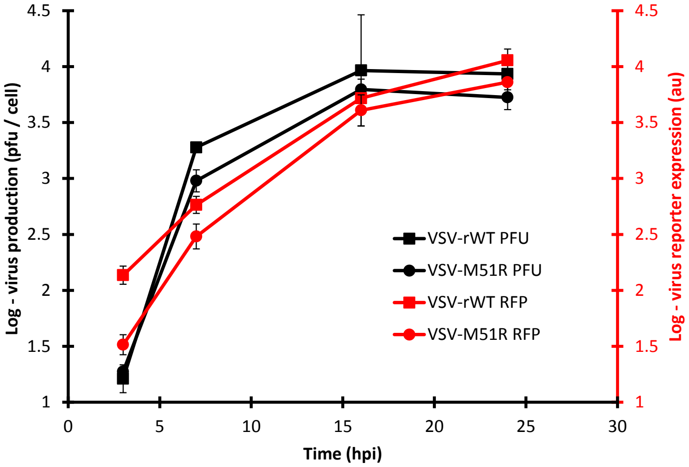

Figure A Plot of RFP and plaque titers at different time post infection for VSV-rWT and VSV-M51R shows similar kinetics and timing of RFP and PFU suggesting RFP as a good surrogate for virus particle production.

The methods used to obtain this data are as follows. One day before infection, PC3 cells were plated in 12-well plates at a density of 1.8 x 105 cells/well. PC3 cells are highly resistant to infection by VSV, so to achieve a synchronous infection 200 µL of stock virus (VSV-rWT, VSV-M51R) was added directly to cell monolayers and allowed to adsorb for one-hour in an incubator, rocking plates every 20 minutes. The virus was titered on PC3 cell monolayers, and based on this number the effective MOI, was approximately 15 (∼ 3500 MOI on permissive BHK cells). After adsorption, the stock solution was removed and the cells were rinsed with sterile PBS and overlaid with 1 ml PC3 infection media. Two other 12 well plates were used for infected and uninfected monolayer controls. At various time-points, these plates were imaged with a Typhoon FLA 9000 (GE Healthcare Life Sciences) and then one-well at each time-point was sacrificed to sample for virus production. The infected and un-infected controls were used to normalize the RFP fluorescent signal from the Typhoon scanner. Images and samples were taken at the times designated in the figures. The host GFP signal could not be resolved using the Typhoon scanner due to a very high background signal.

1.3 Background subtraction and illumination correction

Background subtraction and illumination correction requires dark-field (DF) and illumination-field (IF) images. DF images are images taken with the illumination shutter closed using exposure times that match the exposure times of the images taken in the channels used for quantification. In our case, the green and red channels required the same exposure times so the same set of DF images were used for correction. Many DF images are taken in succession to provide more data for a better estimate of the inherent camera noise. The DF images are broken up into groups of four. Each group of four is used to determine a median pixel intensity for each pixel. The results from each group of four are then averaged to result in a single DF image to be used in subsequent correction steps.

IF images are fluorescence images taken with a fluorescent standard that should result in uniform fluorescent intensity across the entire image if the illumination were to be completely even. Many IF images are obtained for a better estimate of how the sample is illuminated. IF images are taken using fluorescence reference slides (Ted Pella, Inc., item 2273; Redding, CA) at four different locations 25 times each. Again the images are broken into groups of four such that each image in each group is from a different location. The medians from each group are then averaged. By taking the median of the four different locations, imperfections in the fluorescent reference slides are largely removed for more accurate quantification.

Typically, flat-field correction is done using the following process where X represents the image to be corrected, X_{dc} represents the dark-field subtracted image, and α represents the background subtracted and normalized IF image with pixel intensities that represent the correction factors to be applied to the pixels of each experimental image. Thus, Y, the corrected image is created by multiplying X' and α.

$$X_{dc} = (X - DF) \quad (1)$$

$$\alpha = \frac{mean(IF - DF)}{IF - DF} \quad (2)$$

$$Y = \alpha \, X_{dc} \quad (3)$$

In our case, Eq 1, provides insufficient background subtraction for the experimental images prior to multiplication with the correction image, α. This is primarily due to a sigificant and changing level of background flouorescence over time. During the infection, cells lyse, resulting in the release fluorescent protein into the supernatant over time. Further, as cells increase in fluorescence intensity, light emitted from each cell is scattered and produces a diffuse yet significant level of background intensity. Thus, the background intensity changes significantly over time and adds to the fluorescence intensity emitted directly from the cells. Besides the IF and DF image information, we must also eliminate the diffuse background for each image to more accurately quantify the cellular signal of interest. Given the cell emitting the fluorescent signal is significantly smaller in physical size than the diffuse background fluorescence, we can employ a 'rolling-ball' background subtraction to eliminate the diffuse background for each experimental image, X. In actuality, we use the analogous 'sliding-paraboloid' method provided by ImageJ. However, after application of the sliding-paraboloid correction, a histogram of image intensities readily shows that background noise has a non-zero mean given that the sliding-paraboloid algorithm subtracts background based on the local minima instead of the local background mean. Further, the histograms also show that the standard deviation of the noise increases over time, resulting in an increase of the mean of the background noise over time after sliding-parabaloid subtraction. Thus, we face the same problem as before. The noise increases over time and is additive with the signal, so the use of a single correction image, α, for all images produces different levels of correction over time, albeit the differences are now much smaller. To minimize this more subtle effect, the distribution of background intensities in each background corrected image needs to be centered around zero prior to multiplication. To do this, the peak in the histogram in the range of the background noise is determined and the corresponding intensity value is subtracted from the image.

Thus, in total, three background subtraction steps are used prior to multiplication with the correction image, α. First, the dark-field image, DF, is subtracted from the experimental image, X, to obtain X'. A sliding paraboloid is applied to X' to obtain X''. The mode of the background in X'' is then subtracted from the entire image X'' to approximately center the distribution of the background noise about zero to create X'''. The correction image, α, is then multiplied with X''' to obtain a fully background subtracted and illumination corrected image. However, in order to save the image as an 8-, 12-, or 16-bit image, which can only handle numbers ≥ 0, we add a nominal value, β, to the image that is greater than the absolute value of the most negative number in X''' (Eq 4). The value of β is then subtracted from any subsequent measurements taken from that image.

$$Y = \alpha \, X''' + \beta \quad (4)$$

1.4 Cell identification

Signal from Hoechst nuclear stain is used to locate individual cells at each time point. Given that thousands of cells are tracked for hundreds of time points in multiple wells, automated nucleus identification is required to make the approach feasible. Nuclear locations are determined using the method provided by ImageJ in the 'Find Maxima' function. Prior to use of the 'Find Maxima' function, the nuclear staining images are filtered using a mean filter with a radius approximately equal to half the radius of the nuclei to make the image of each nucleus appear to be a smooth and rounded feature with a single maximum to avoid spurious local maxima while maintaining the ability to discriminate adjacent nuclei. Documentation for the 'Find Maxima' function is available from ImageJ but the function as it is implemented in JEX (function name: Find Maxima in One Color) takes two parameters, a threshold and a tolerance. The threshold is the minimum intensity that can be considered a maximum while the tolerance is the minimum drop in intensity that must exist between adjacent local maxima (*i.e.*, the depth of the valley between two peaks necessary) to produce two separate maxima instead of ignoring the lesser of the two and producing only one maximum. The locations of nuclei determined by the algorithm are checked visually to ensure high accuracy in identification. JEX then applies the algorithm uniformly to each image in the dataset to objectively identify cells at each timepoint.

2 Baseline FC and Microscopy experiments

2.1 Cell preparation

PC3 IFIT2-ZsGreen reporter cells cultured to 90% confluency, were harvested by trypsinazation, diluted to 3.0x10^5 cells/ml, and plated into 12 well tissue culture plates at 1 ml/well. Cells were incubated for 24 hours to adhere and grow, after which they were 80-90% confluent. Approximately 18 hr prior to harvest, the imaging plate along with parallel sampling plates were infected with an effective MOI of 5-10 of either VSV-rWT or VSV-M51R or mock infected. Virus inoculum was absorbed for 20 min at 37°C, 5%CO^2, after which it was removed and cells were rinsed 1x with DPBS. 1 ml of RPMI 2% FBS containing Hoechst 33342 (Anaspec #83218) diluted 1:20,000 as a live-cell nuclear stain was added to cells. At the appropriate timepoint, cells were harvested by trypsinization, fixed with PFA 4% for 20

min, and resuspended in RPMI 2% FBS, and transferred to flow tubes.

2.2 Data acquisition and analysis

Microscopy - Imaging for baseline microscopy experiments was performed on a Nikon Eclipse-7980 Ti fitted with an outer warming chamber set to 37°C and a stage-top chamber (both from InVivo Scientific) set to 5%CO_2, with humidification. Fluorescence illumination was provided by a Lumencor SpectraX Light Engine and Chroma filters. Images were acquired with a Hamamatsu C11440-22CU and stage movement automated by an ASI MS-2000. Nikon NIS Elements was used to automate imaging.

A 2-by-2 array of images in 3 colors (R,G, and B) at 10X magnification was captured for each condition at each timepoint. In order to make more quantitative measurements, images are first background subtracted and corrected for any uneven illumination using a refined approach based on standard flat-field correction (see 'Quantification of single-cell fluorescence'). The locations of nuclei are determined from the images of the live-cell Hoechst nuclear staining as described above. Mean intensity within a small radius around the nuclear locations is quantified in each channel of the image set. The diameter of the circle is chosen to be slightly smaller than the large majority of nuclei. All baseline microscopy single-cell analysis was performed using simple 8 pixel diameter (circular) ROIs positioned at the nucleus of each cell. The small diameter reduces quantification outside the cell identified by the nuclear signal yet is large enough to average over pixel-to-pixel noise to produce a reliable measure. The nominal value β from Eq 4 is subtracted from each measurement and the result stored in a data table.

FC - FC data was acquired on a BD LSR II equipped with a 488nm laser to excite ZsGreen, a 561nm laser to excite DsRed, a UV lamp to excite Hoechst 33342, with appropriate filters for each channel. Cells were first gated on FSC/SSC to discriminate between whole cells and debris and then on the Hoechst signal to target single cells. A minimum of 10,000 single cells per sample were analyzed.

In order to better compare the traditional FACS data of our reporter system experiments with the image cytometry data resulting from our time lapse microscopy we needed to be able to perform analysis on a similar platform. However, most flow cytometry specific software packages such as FlowJo and FCS-Express can only read .fcs type files, to which our image cytometry data could not easily be converted. Fortunately, the statistical package R has several libraries (flowcore, flowviz available from the CRAN online repository) designed for the analysis of flow type data which can both import and plot .fcs files and also analyze and plot similar data tables, such as those resulting from our image quantification. Therefore, R provided a uniform platform on which we could analyze both data types. We first used FlowJo to do basic forward scatter/side scatter gating on the FC data to eliminate debris and large clumps of cells from the subsequent analysis. The data after these steps was extracted/imported into R data tables, and plotted in an analogous manner to the microscopy data for direct comparison.

2.3 Mock infection data

See Fig. B for scatter plots of mock infected controls from baseline FC and microscopy experiments.

2.4 Earliest timepoint data

The 6 hour timepoint is the earliest timepoint for FC experiments and is plotted in the main manuscript while the microscopy data began 2.83 hpi and is plotted in Figure C.

2.5 Population Dynamic Cytometry Plots and Movies

Population dynamic cytometry plots (PDC plots) for each condition of baseline microscopy experiments are available for download with this manuscript as SI files (S3, S4, S5) along with a representative timelapse movie (S2).

3 MA-based IDC experiments

3.1 Cell preparation

PC3-IFIT2 reporter cells were infected in solution with either the VSV-M51R or the VSV-rWT virus strains at a effective multiplicity of infection (MOI) of 10. The methods for determining MOI and performing the in solution infections have been previously described.[1] Following the virus adsorption period, the temperatures of the virus-cell solutions were raised to 37 °C in a water bath for 7 min to allow for internalization of the attached virus. To remove any excess virus, the cell solutions were centrifuged (1000 rpm, 4 min), the infection media decanted, and the infected cell pellet re-suspended in fresh media three times. With the final re-suspension, the cell density was adjusted to the optimal density for microwell seeding (1-2 x 10^5 cells/mL). The re-suspension RPMI media contained Hoechst 33342 (AnaSpec, 1µM) and HEPES (Sigma-Aldrich, 25 mM). Hoechst 33342 is a live-cell nucleic acid stain that can be used to identify the location and number of cells in microwells. HEPES is a buffer commonly used in microfluidics applications to protect the cells during timelapse microscopy.

MA cell preparation differed from that used for microscopy experiments. Virus in MA experiments was adsorbed to suspended cells on ice to prevent entry of accumulated virus on the surface of the cell until the media was warmed over a short period just prior to imaging. This was done to minimize the time between the start of infection and the start of imaging, which consists of 20-30 min of time for seeding and sealing the MA device and microscopy

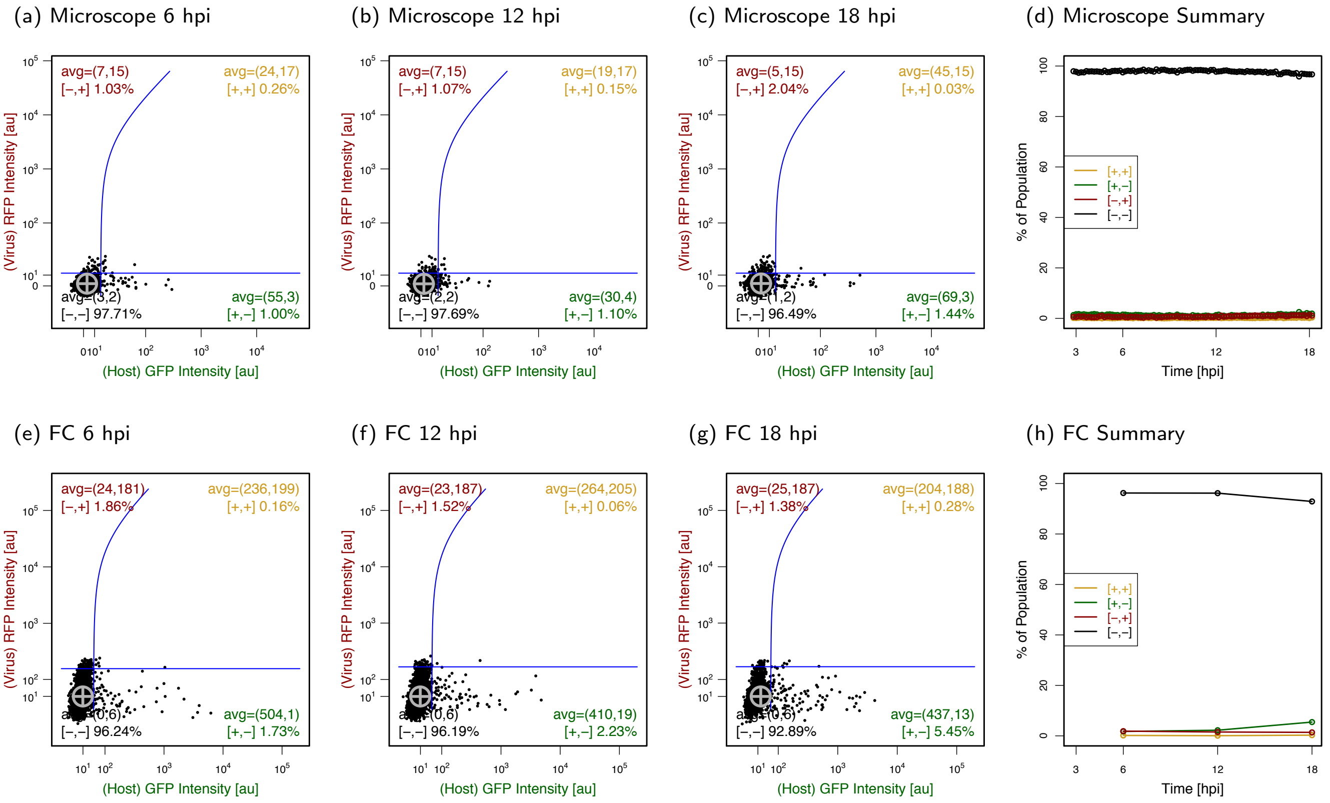

Figure B Movie frames of the PDC plots of mock infected controls from baseline experiments at 6, 12, and 18 hpi as well as a summary plot of gated population percentages and means for each timepoint.

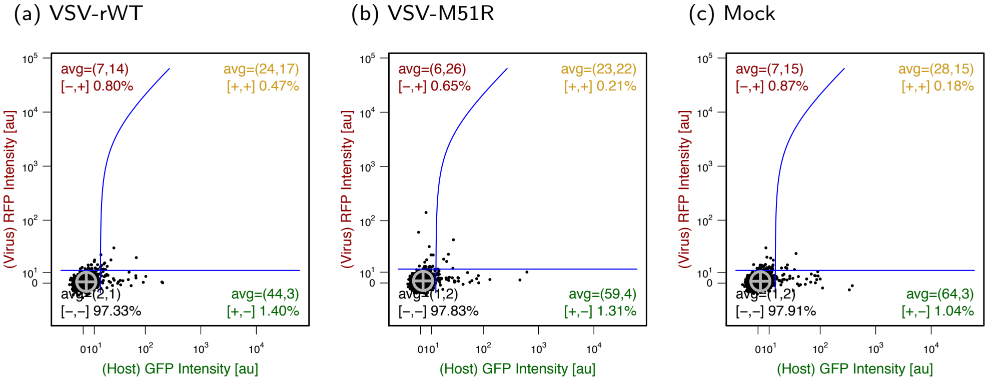

Figure C PDC plots for microscope (standard culture) experiments at earliest timepoint of 2.83 [hpi].

setup. Virus in microscopy experiments was incubated with adhered cells at 37 °C for 20 min and washed away prior to microscope setup. This allowed viral entry during particle adsorption, resulting in a potentially less synchronized start to infection compared to MA expeirments. Furthermore, different infection efficiencies are associated with different protocols[1], thus raw viral titers (virus-per-unit-volume) were adjusted to produce the same effective MOI (5-10) for baseline and MA experiments.

3.2 Bull's-eye device fabrication

The bull's-eye device is constructed using two microscope slides and a polydimethylsiloxane (PDMS) bull's-eye microwell array molded from a master created via soft-lithography techniques which have been discussed elsewhere[2,3]. The microwells are designed to be 50 µm × 50 µm × 50 µm with a 50 µm space between the edges of each well and are similar to that used by others in the literature[4,5]. The array is propagated over the area of a 1 in × 3 in microscope slide. Superimposed on the microwell array

are recessed regions, each with the shape of a bull's-eye. The bull's-eye design was developed to isolate different regions of the microwell array for independent pipette operations without having to cut or break apart the device. 12 bull's-eye designs fit on the microscope slide array to allow for 12 different experimental conditions per microscope slide (although only 10 per slide were used in conjunction with the clamp apparatus). Each bull's-eye contains $\sim$ 2500 microwells. The outer-ring of each bull's-eye pattern has an inner diameter and width of 6 mm and 1 mm and is referred to as a moat. The recessed region at the center of each bull's-eye has a diameter of 1.5 mm. The moats and centers are recessed approximately 0.4 mm from the top surface of the microwell array.

3.3 Device preparation and seeding

Approximately 1 hour before beginning the infection procedure, the PDMS device is placed under a UV lamp for sterilization. After 20-30 min, the device is moved to a vacuum chamber for de-gassing. This step is critical, as it allows liquid to readily fill the wells instead of trapping air underneath the liquid layer. After 20 min in the vacuum chamber, 100 µL droplets of infection media (*i.e.*, media containing 2% FBS instead of 10% FBS) with HEPES and Hoechst but no virus are placed on each bull's-eye to wet the device. There is sufficient surface tension to keep the droplets from spilling into the moats. The PDMS device is placed in an incubator, with the droplets in place before beginning the infection procedure. The device is in this humid environment for approximately 1 hr before cell seeding begins.

To seed the infected cells into the microwell device, the existing droplets are removed and replaced with 70-80 µL droplets of the infected cell solution (1-2 x 10^5 cells/mL). After 30-60 s, the droplets are removed by placing the pipette tip in the recessed center and aspirating quickly, followed shortly by a gentle dispensation of fresh media to avoid evaporation. The swift removal of the droplets sweeps cells off the top surface of the device, but does not disturb the cells that have settled into the microwells. The bull's-eyes are washed with fresh media once or twice more in the same manner. The device is sealed by quickly removing the droplets from the bull's-eyes and gently pressing on a glass slide (the top outward facing side treated with tween to avoid droplet formation from condensation during timelapse microscopy). Pressure is applied using a clamp apparatus to maintain a seal between microwells as described in 'Bull's-eye device fabrication and assembly'.

3.4 Clamp assembly

Dimensions and notes regarding the design of the clamp apparatus are contained in Fig. D & E.

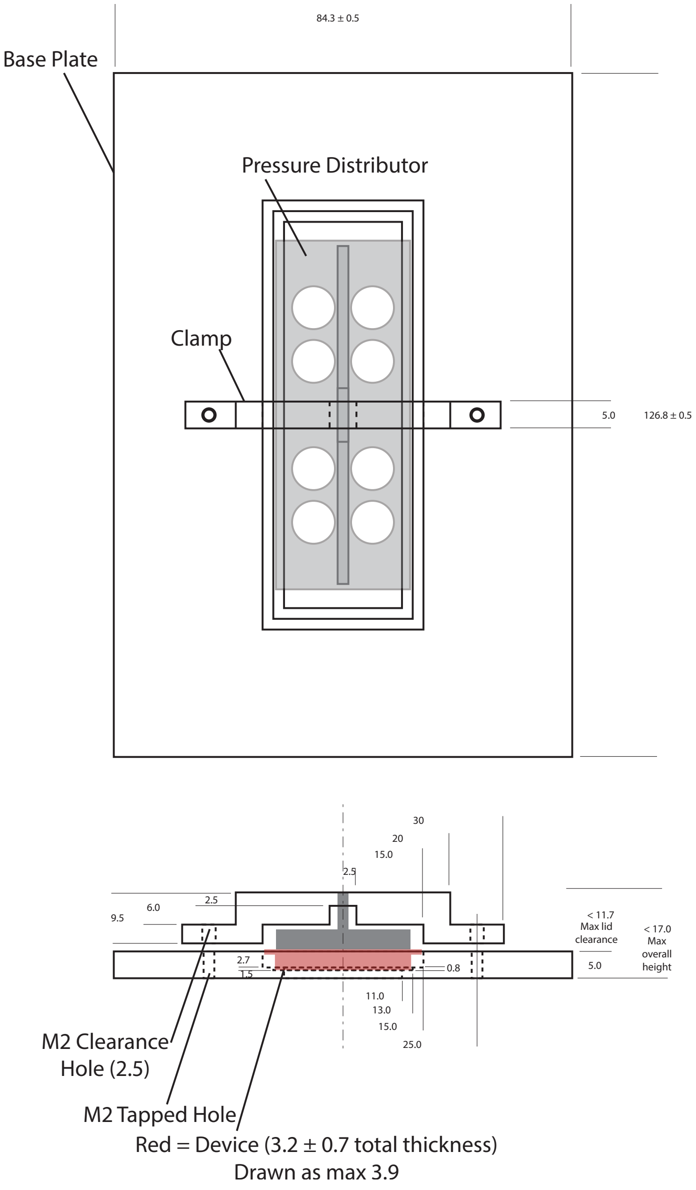

Figure D Bottom piece to MA clamp apparatus with footprint of a 96-well plate to fit on a microscope stage.

3.5 Data acquisition and analysis

Imaging was performed using a microscope equipped with the following components to enable fluorescence time-lapse microscopy of live cells. Microscope body: Nikon TE Eclipse 300. Outer warming chamber: InVivo Scientific, set to 37°C. Stage-top incubator: Pathology Devices, set to 37°C, 5%CO_2, and 80% RH. Illumination: Chroma PhotoFluor. Automated filters: Lambda 10-2. Automated stage: Prior ProScanII. Imaging software: MetaMorph v.7.7.8. During imaging, Kim wipes (Kimberly-Clark) soaked in sterile PBS were placed within the slide holder apparatus to increase humidity immediately surrounding the device. A 3-by-4 array of images in 3 colors for each bull's-eye was acquired every 30 min at 4X magnification (1.6125 pixel/µm).

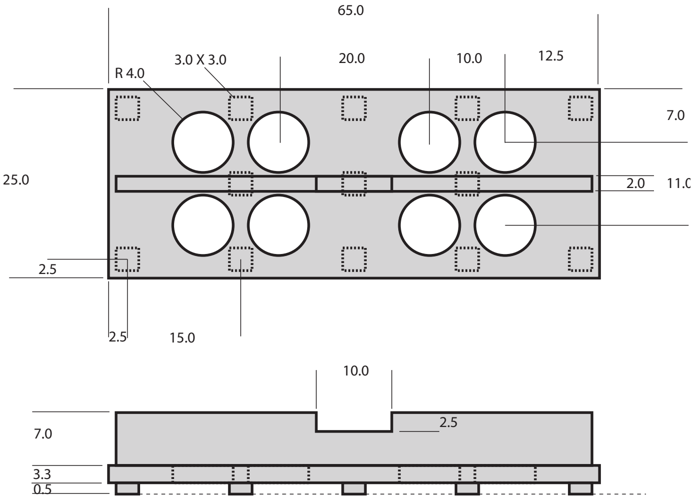

Figure E Top piece to MA clamp apparatus for distributing clamp pressure evenly over the MA.

Automatic microwell identification - Microwells are identified using two phases. The first is to identify potential locations of microwells, while the second uses the most promising locations as seed points for building a grid of potential locations that matches what is expected in the image. The potential locations are determined via convolution with an image kernel. The kernel is a square with a border. The center of the square and the border can be defined as being either white (a value of 1) or black (a value of 0). The overall width and height of the square, as well as the border width, and colors are defined by the user based on what will best convolve with either the bright-field/phase-contrast or fluorescent image. Typically a kernel with a black center and white border is used to with a bright-field image while a white center and black border is used with a fluorescent image. When using bright-field, convolution results are typically enhanced when the bright-field images are first processed using a edge-detection filter of some sort to make the microwell boundaries appear brighter than the centers or surrounding regions of the microwells.

The convolution phase (function name: Microwell Convolver) produces an image where bright peaks indicate likely locations of microwell centers and is used in the second phase of building a fully connected grid of points (*i.e.*, a potentially non-rectangular grid in which each location is connected to at least one neighbor) representing microwell centers. To determine which peaks are actually microwells we use the function named 'Microwell Finder'. In this function, the ImageJ 'Find Maxima' function is first leveraged to determine the locations of the peaks in the convolution image using only 1 defined image channel of the multi-channel image set. The result of the algorithm organizes the points according to their peak heights, which means the first points in the list are the locations with the highest convolution score and thus highest confidence of being the center of a microwell. Beginning with the first point in the list, a grid is built based on user specifications regarding the expected spacing and potential deviations from horizontal and vertical. If a grid of sufficient size, as indicated by the user, cannot be built according to the specifications from the first point, the second point in the list is used as the seed point for attempting again. The grid building phase increases the robustness and success rate of automatically determining microwell locations immensely, allowing the user to use a single set of parameters to determine microwell locations for an entire set of bull's-eyes on a microscope slide. On the occasion that the settings do not succeed in finding the microwell centers for a particular group of images, JEX allows one to re-run the algorithm with different parameters on just those subsets of images.

Additional options to the Microwell Finder function enable the ability to avoid identifying microwells on the borders of the images (*i.e.*, those that may not be fully captured in the image) as well as any microwells that are on the border of the grid. This is useful for allowing the images to be processed and analyzed without having to stitch the images first. In other words, if images are taken that have a small amount of overlap, microwells on the borders of images have the potential to be quantified twice, once in one image and again in the overlapping image. By eliminating wells on the border this possibility is eliminated. Some microwells might go un-analyzed; however, this is preferred to producing 'duplicate' data points. Given the dimensions of the bull's-eye pattern and microwells, there are $\sim$ 2500 microwells per bull's-eye. Using this approach we typically identify $\sim$ 2200 microwells per bull's-eye, which is able to provide sufficient data for this study and general approach

Quantification of whole cell intensity - Using similar approaches described for baseline microscopy experiments, images were corrected and stitched and cells were identified. The algorithm used to quantify fluorescence from a microwell containing a single cell was optimized to increase signal to noise (SNR). Instead of using the integrated intensity from the entire microwell region of interest as the signal, we first convolve the entire image with a circular mean-filter 26 pixels diameter, which is large compared to the cell ($\sim$16 pixel diameter), but is smaller than the microwell width (33 pixels). We then find the maximum intensity within the microwell *in the mean-filtered image*. Furthermore, we limit the search for this maximum to a region slightly smaller than the microwell given the peak intensity of a cell is generally at least 1 cell-radius inside the microwell boundary. This restriction reduces the chance of quantifying signal from an adjacent microwell (*e.g.*, scattered light from a particularly bright cell). We then multiply the max of mean-filtered mi-

crowell region by the area of the mean filter to convert the measure to integrated intensity of the cell (and background, which will be subsequently subtracted). This approach provides a relatively simple means to dynamically 'position' an ROI to maximize the amount of intensity collected from each singly isolated cell. Use of an ROI that encompasses the microwell to quantify fluorescence in the microwell includes unnecessary background pixels in the measurement which significantly reduces the SNR (details to follow). The approach utilizing the radial mean filter is only appropriate to use when a single cell is contained within the microwell. Using an ROI to encompass the entire microwell is more general and can be used in other cases to sum the signal from multiple cells contained in a microwell. Since the cell takes up a minor fraction of the microwell region, the mode of the intensity histogram of the unfiltered microwell image is used to measure the background intensity for background subtraction.

The increase in SNR using this method can be seen mathematically as follows. However, it should be noted that the following analysis is not meant as an absolutely rigorous analytical determination of system behavior but an appropriate means to estimate and illustrate the influence of restricting the ROI to exclude extraneous background pixels.

For each measured region of interest (ROI), made up of n_{tot} pixels, we consider the special case where n_{sig} of the n_{tot} pixels contain a signal with a mean intensity of I_{sig} and the remaining pixels, numbering n_{bg}, contain background noise having an intensity of I_{bg}. (Eq 5).

$$n_{tot} = n_{sig} + n_{bg} \tag{5}$$

The pixel-to-pixel noise in the intensity of the background pixels, $\sigma_{bg,p2p}$ is assumed to be a constant value of β, while the noise for the signal pixels, $\sigma_{sig,p2p}$, consists of both background noise and signal noise and depends upon characteristics of the camera, which are summarized into the constant parameter α. Although Poisson in nature, the noise within the signal and background pixels approach normal distributions for intensities typical of our study. Each of these pixel-to-pixel standard deviations, when measured over n_{sig} and n_{bg} pixels have a measurement standard deviation of σ_{sig} and σ_{bg}, respectively. (Eqs 6-9).

$$\sigma_{bg,p2p} = \beta \tag{6}$$

$$\sigma_{bg} = \frac{\beta}{\sqrt{n_{bg}}} \tag{7}$$

$$\sigma_{sig,p2p} = \alpha\sqrt{I} + \beta \tag{8}$$

$$\sigma_{sig} = \frac{\alpha\sqrt{I} + \beta}{\sqrt{n_{sig}}} \tag{9}$$

Therefore, Eq 10 represents the mean intensity $\pm\sigma$, while Eq 11 represents the signal-to-noise ratio (SNR).

$$\text{Mean Intensity} = (n_{sig}I_{sig} + n_{tot}I_{bg})/n_{tot} \pm \sqrt{\sigma_{sig}^2 + \sigma_{bg}^2} \tag{10}$$

$$SNR = \frac{(n_{sig}I_{sig} + n_{bg}I_{bg})/n_{tot}}{\sqrt{\sigma_{sig}^2 + \sigma_{bg}^2}} \tag{11}$$

Eq 11 is used in Fig. F to plot the SNR of the region-of-interest (ROI) normalized to its maximum SNR possible (*i.e.*, when all the pixels are comprised of signal pixels and none are purely background signal). This normalized SNR is plotted for different ratios of n_{sig}/n_{tot}. Due to normalization and the assumption that I_{bg} has a mean of zero, this plot is insensitive to the intensity being measured, I_{sig}, but still depends upon α and β. In our system, β was approximately 20 while α was approximately 1.35 (See discussion surrounding Eq 13). The plot shows that SNR characteristics of the measurement from a microwell depend upon how many extraneous background pixels are contained within a region of interest compared to the pixels that contain signal. The equation suggests that the SNR is increased by a factor 3.6 by changing from a 33 × 33 pixel rectangular ROI (the size of the microwell) to a circular ROI with radius of 13 pixels like that used in this study. This calculation agrees well with observations during development of the approach (data not shown). If the diameter of the mean-filter were to be reduced to 11 pixels in diameter instead of 13 pixels, the SNR ratio improvements becomes a factor of 6.3; however, reducing the size of the mean-filter increases the risk of failing to encompass the entire cell due to asymmetric cell morphology. Typically removal of extraneous background pixels is done by image segmentation, which has its own challenges. Thus, the advantage of this approach is increased SNR without the need to segment the image and potentially introduce associated artifacts into analysis.

The signal and background intensities are recorded in a data table and used in subsequent analysis within R. The following numbered list describes each subsequent step of data analysis.

Analysis in R

1. Data is loaded regarding the cell locations (from JEX), microwell intensities (signal and background measures as just described from JEX), and a list of microwell IDs in which experimental errors are suspected, such as microwells with bubbles in them (acquired via manual curation prior to analysis).
2. Microwells identified with potential errors are removed.
3. Convert frame number to timestamp.
4. Calculate background corrected values for each color (i.e., max - mode).
5. Get the mean signal from wells will 0 cells, termed null wells, over time for each ROI and take the median of those values for the given image.
6. Subtract the median signal of null wells obtained for each image from all microwell data in the corresponding images

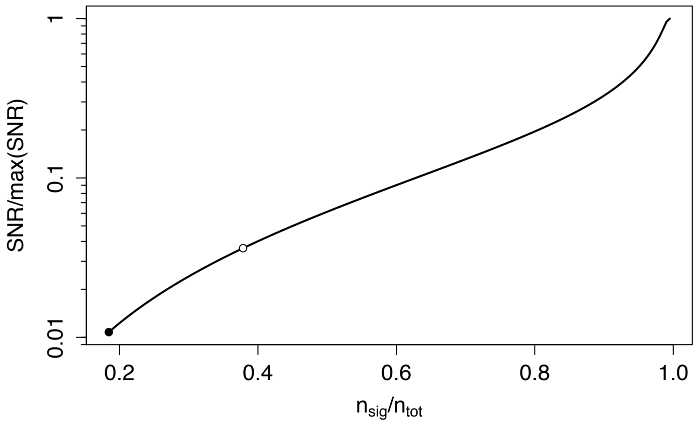

Figure F SNR charactersitics for different ROIs during measurement of single-cell fluorescence. n_{tot} represents the number of pixels total in the ROI while n_{sig} represents the number of pixels that contain detectable signal. The remaining pixels exhibit background noise. Black dot - SNR when using the entire microwell region (33 × 33 pixels). White dot - SNR for the 13 pixel radius circle used in our approach is shown as a white dot. Locations of dots assume the cell signal was contained within a spot with an 8 pixel radius (*i.e.*, $n_{sig} = \pi(8)^2$) and that the mean background intensity is 0, $\alpha = 1.35$, $\beta = 20$.

7. Apply illumination correction
8. Separate the data into data for 0-cell wells and 1-cell wells
9. Determine the threshold for detection based on 0-cell well data (*i.e.*, median + 3σ).
10. Set to 0 any timpoints in each trajectory below threshold and any spurrious points above threshold before consistent signal (*i.e.*, when less than 4 points in a row are above the detection limit).
11. For each color, mark trajectories that have a signal using a boolean flag.
12. Write the preprocessed data to a file.

Following pre-processing, analysis of the data is performed and described below.

1. Read in the preprocessed data.
2. Fit the first 4 detectable timepoints with an exponential growth curve (see section 'Curve Fit Determination of α & τ').
3. Save flags for each trajectory that indicate if the R^2 value for the fit was above 0.9 and if the production rate parameter, α, was greater than 0. Set fit quality flags to indicate trajectories for which *alpha* was above 0 *and* R^2 was greater than 0.9.
4. Determine other kinetic parameters described in Fig. 6 of the main manuscript such as delay-times, rise-times, max-intensities, and cell lysis.
5. Write the summary of all of these parameters for 1-cell wells to a file.

3.6 Curve Fit Determination of α & τ

Single-cell fluorescence intensity measurements over time (fluorescence trajectories) were fit with an exponential equation of the form shown in Eq 12 to determine α, the intrinsic growth rate of the curve, and τ, the estimated delay-time prior to significant exponential growth. The curve fit is performed on the first 4 timepoints of above the limit of detection to determine these parameters.

$$y = e^{\alpha(t-\tau)} \tag{12}$$

However, it should be noted that noise in microscopy data typically is not constant for all intensities; thus, it is important to account for the different noise levels during the fitting process. The majority of pixel-to-pixel noise in the image follows a Poisson distribution and is a function of the number of photons detected.[6] The standard deviation of the number of photons reaching each pixel is equal to the square root of the mean number of photons. Using this observation we can weight each data point appropriately to avoid over fitting to points with increased noise, or vice versa. The changing uncertainty is accounted for in least-squares fitting by weighting the points inversely proportional to the expected variance (i.e., $1/\sigma^2$). However, our method uses the mean of $n \approx 530$ pixels to quantify intensity. Thus, relative to pixel-to-pixel noise, the magnitude of the noise in the measurement should be reduced by a factor of $\sim 1/\sqrt{n}$. We use Eq 13 to estimate the Poisson noise, σ_p, where i is the intensity measurement (including background from the raw image) obtained from an average of n pixels, 18000 [electrons] is the full well capacity of the camera, 0.6 [electrons/photon] is the typical camera quantum efficiency, and 16384 [intensity units] is the dynamic range of the camera.[6] Using this equation, we expect σ_p to be ~ 1 photon or ~ 0.6 intensity units for our camera and quantification method.

$$\sigma_p \approx \frac{1}{\sqrt{n}}\sqrt{\frac{18000/0.6}{16384} \times i} \approx 0.059\sqrt{i} \tag{13}$$

When we look at the data measurements we see a similar estimate. The first few time points of data in the red channel (i.e., when no cells are expressing red) suggest that the initial noise ranges from 0.16 to 1.16 across different images with a mean of ~ 0.7 intensity units. Thus, it appears that the primary source of noise is indeed initially Poisson noise justifying our use of the relationship between the mean intensity and standard deviation to weight each datapoint for fitting. In this case the equation for the weight, w, simplifies to $w = 1/i$.

We use the publicly available ‘drc’ package for R to performing the non-linear least-squares (NLLS) fits.[7] NLLS fitting does not guarantee an optimal fit of the data and can only do so with an appropriate initial guess for the parameters. To address this, we provide the package a function to determine an initial guess. The function takes the natural log of the data and performs a linear fit, which is guaranteed to find an ‘optimal’ fit to the transformed data, to get an estimate of α and τ for NLLS fitting. We use the NLLS fit method for the final estimate because the linear fit inappropriately weights the data points due to the log transformation, inducing non-normal noise in the data. Thus, the linear estimates are flawed but are close enough to aid in performing robust non-linear estimates.

3.7 Quantification of cell lysis

Lysis was detected as the first timepoint that dropped to below 60% of the average of the previous 5 timepoints. The method of quantification used for the microwells is sensitive to lysis because the fluorescence signal intensity is always measured relative to the mode of the histogram taken from the entire microwell. Prior to lysis, the fluorescence signal coming from the microwell is contained within the cell so the signal detected in the microwell is significantly different from the microwell background intensity (i.e., the mode). Therefore, when the cell lyses and the fluorescence intensity becomes evenly distributed throughout the microwell, the signal detected in the microwell and mode of the histogram become nearly the same, producing a difference of ~ 0, which is then seen as a precipitous drop in the measured signal over background coming from the microwell.

We have also provided an example of a timelapse movie (S6) created from microwell image data that illustrates examples of the cell lysis and demonstrates that each microwell is sealed from the others.

For the specific purpose of examining cell lysis, we also manually code each trajectory to indicate the overall shape of the curve (0 = No Plateau Verified, 1 = Plateau, 2 = Lysis, 3 = Neither).

3.8 Earliest timepoint data

For microwell experiments, the earliest timepoints was 1.42 hpi which is plotted in Fig. G.

3.9 Population Dynamic Cytometry Plots and Movies

Population dynamic cytometry plots (PDC plots) for each condition of microwell experiments are available for download with this manuscript as SI files (S7, S8) along with a representative timelapse movie (S6).

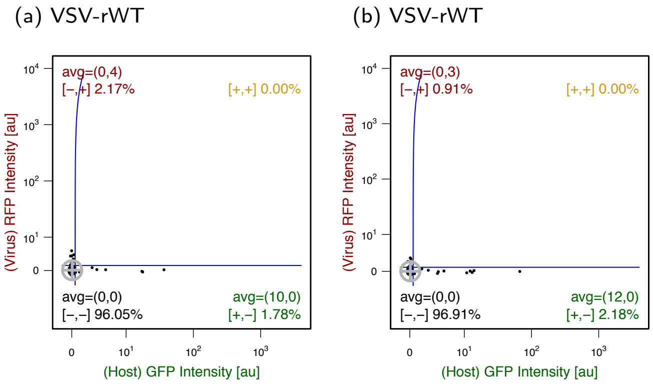

Figure G PDC plots of microwell data at earliest imaged timepoint of 1.42 [hpi].

3.10 Example JEX database and microwell image analysis

Here are the steps to use JEX to load, process, and view an example dataset dedicated to this manuscript which is hosted online at SourceForge.net.

1. Go to http://sourceforge.net/projects/jex-example/ and download ‘JEX Example.zip’
2. Unzip the contents of the file, creating a folder called ‘JEX Example’ that contains folders and files named ‘Example Database’, ‘Example Workflow.jwf’, ‘Raw DF Images’, ‘Raw IF Images’, and ‘Raw Microwell Images’.
3. Go to https://github.com/jaywarrick/JEX/wiki.
4. Read the wiki page called ‘Home’ along with reading and following the instructions on wiki pages 01 (How to Download JEX, version 0.0.9) and page 02 (Getting Started) up through step 2 of creating a JEX repository. When creating the repository, choose the ‘JEX Example’ folder you created in the previous step. This folder contains a JEX database already. Therefore, by pointing JEX to this folder, it will recognize the database and show it in the list of databases under the repository header you just created.
5. Once the database is recognized and shows in the list, you can then click *on the database icon* to open the database titled ‘Example Database’. The database will contain no image data yet.
6. Read wiki page 08 (Save and Load a Workflow) to see how to load a JEX workflow.
7. Load the JEX workflow file called ‘Example Workflow’ that is contained within the ‘Example Data’ folder.
8. Click the white square just above the ‘BROWSE/SELECT OBJECTS’ header on the left panel of JEX. This should turn the box red. and

indicates that this entry within the Dataset is selected (read further wiki pages for more details on Datasets, Entries, and Entry selection if desired).

9. Click tab #5 labeled “Process” (located along top edge of the JEX window)

10. The JEX workflow you have loaded has three functions at the beginning that import images (function name: Import Images (SCIFIO)). We must set the input directory of each of these functions to the appropriate folder. Click the header/name of the first function panel. This will trigger JEX to show the parameters of this function in the lower middle-right panel of JEX.

11. Click the button labeled ‘...’ next to the first parameter titled ‘Input Directory/File’ to open a file-chooser dialog. Choose, the *folder* ‘<wherever you saved the folder>/Example Data/Raw Microwell Images’.

12. Repeat the previous two steps for the second and third functions choosing the ‘<wherever you saved the folder>/Example Data/Raw DF Images’ and ‘<wherever you saved the folder>/Example Data/Raw IF Images’, respectively.

13. Click the large ‘Run’ button to run the workflow that you have loaded. This will run all the functions and save a copy of the workflow into the database to save the precise parameters that you ran. The workflow loads the raw data and processes it to generate fluorescence data for cells in microwells as well as ROIs and the (IF-DF) image for illumination correction.

Double-clicking the database object icons will allow you to view the data. If it is an image, a multi-dimensional image viewer will open. If it is a .arff file (i.e., the file type used to save the tabular data), a table viewer will open. If you click on an ROI object, nothing with happen. Instead, open an image and within the image viewer you can view the ROI object overlaid on the image. Other file types will open in the computers default application for that file extension.

References

1. Timm A, Yin J (2012) Kinetics of virus production from single cells. Virology 424: 11-7.

2. Duffy DC, McDonald JC, Schueller OJA, Whitesides GM (1998) Rapid prototyping of microfluidic systems in poly(dimethylsiloxane). Analytical Chemistry 70: 4974–4984.

3. Sia SK, Whitesides GM (2003) Microfluidic devices fabricated in poly(dimethylsiloxane) for biological studies. Electrophoresis 24: 3563–3576.

4. Adalsteinsson VA, Tahirova N, Tallapragada N, Yao X, Campion L, et al. (2013) Single cells from human primary colorectal tumors exhibit polyfunctional heterogeneity in secretions of elr+ cxc chemokines. Integr Biol (Camb) 5: 1272-81.

5. Gong Y, Ogunniyi AO, Love JC (2010) Massively parallel detection of gene expression in single cells using subnanolitre wells. Lab Chip 10: 2334-7.

6. Waters JC (2009) Accuracy and precision in quantitative fluorescence microscopy. J Cell Biol 185: 1135-48.

7. Ritz C, Streibig JC (2005) Bioassay analysis using r. Journal of Statistical Software 12.
